# Application and Adaptation of Arc-Based Welding Processes to Wire Arc Additive Manufacturing: A Review

**DOI:** 10.3390/ma19204362

**Published:** 2026-10-16

**Authors:** Ahmed Nabil Elalem, Husam Alrehaili, Mohammad Azad Alam, Sufian Raja, Azim Uddin

**Affiliations:** 1 Department of Mechanical Engineering, Wayne State University, Detroit, MI 48202, USA; 2 Department of Mechanical Engineering, Faculty of Engineering, University of Tabuk, Tabuk 71491, Saudi Arabia; hmalrehaili@ut.edu.sa (H.A.); mz-alam@ut.edu.sa (M.A.A.); 3 Daou (Shaoxing) Technology Co., Ltd., East Zone, Keqiao International Park, No. 199 Chuangyi Road, Keqiao District, Shaoxing 312030, China; sufianraja189@gmail.com; 4 Institute for Nanotechnology and Water Sustainability, College of Science, Engineering and Technology, University of South Africa, Florida, Johannesburg 1709, South Africa

**Keywords:** wire arc additive manufacturing, gas metal arc welding, gas tungsten arc welding, plasma arc welding, cold metal transfer, solidification, microstructural evolution, heat input, residual stress, porosity, defect formation

## Abstract

Wire Arc Additive Manufacturing (WAAM) has emerged as one of the most industrially scalable arc-based metal additive manufacturing (AM) technologies, using established Gas Metal Arc Welding (GMAW), Gas Tungsten Arc Welding (GTAW), and Plasma Arc Welding (PAW) processes to deposit metallic material layer-by-layer into near-net-shape components. This review presents a physics-based examination of the adaptation of traditional welding processes for AM applications, with emphasis on: (i) the thermodynamic and metallurgical principles governing melt pool formation, solidification, and solid-state transformations; (ii) the thermal and solidification features that WAAM shares with multi-pass welding and those that are distinctive to WAAM, with particular attention to the temperature gradient-to-solidification-rate ratio (*G*/*R*) and its role in controlling grain morphology; (iii) deposition path strategies and their influence on heat distribution and microstructural homogeneity; (iv) arc metal transfer modes and their thermal implications for AM; and (v) mechanisms of defect formation including porosity, solidification cracking, residual stress, and geometric deviation. Unlike general WAAM reviews, this article specifically positions conventional arc welding processes as the metallurgical and thermal foundation of WAAM and critically examines how their operating principles are transformed under repeated layer-by-layer deposition. Key governing equations linking process parameters to microstructure and properties are presented. Among GMAW variants, Cold Metal Transfer (CMT) provides low heat input, controlled droplet transfer, and low spatter, whereas GTAW and PAW allow wire feed and arc power to be set independently; the preferred process therefore depends on the alloy, required deposition rate, geometry, and surface quality. A synthesis of findings across ferrous and non-ferrous alloy systems is presented, together with knowledge gaps and future directions toward closed-loop adaptive WAAM systems.

## 1. Introduction

Metal additive manufacturing (AM) has reshaped the design, prototyping, and production landscape for complex metallic components across aerospace, defense, energy, automotive, and biomedical sectors. Unlike subtractive manufacturing processes that begin with bulk material and remove material to reveal a final geometry, AM processes build components layer-by-layer directly from digital models, offering substantial reductions in material waste, tooling costs, and lead times, alongside greater design freedom for topology-optimized and internally featured structures [1,2,3].

Within the broad family of metal AM processes encompassing Laser Powder Bed Fusion (LPBF), Directed Energy Deposition (DED), binder jetting, and sheet lamination, Wire Arc Additive Manufacturing (WAAM) occupies a distinctive and growing niche defined by its use of electric arc welding as the heat source and metallic wire as feedstock. WAAM is a DED-family process in the ASTM/ISO classification framework, and its defining advantage over powder-based AM is the combination of high deposition rate (1–10 kg/h), near-100% material utilization, and industrial scalability to component dimensions of several meters, capabilities that remain difficult and economically impractical for most powder-bed processes [2,3,4,5,6,7].

**Figure 1:**
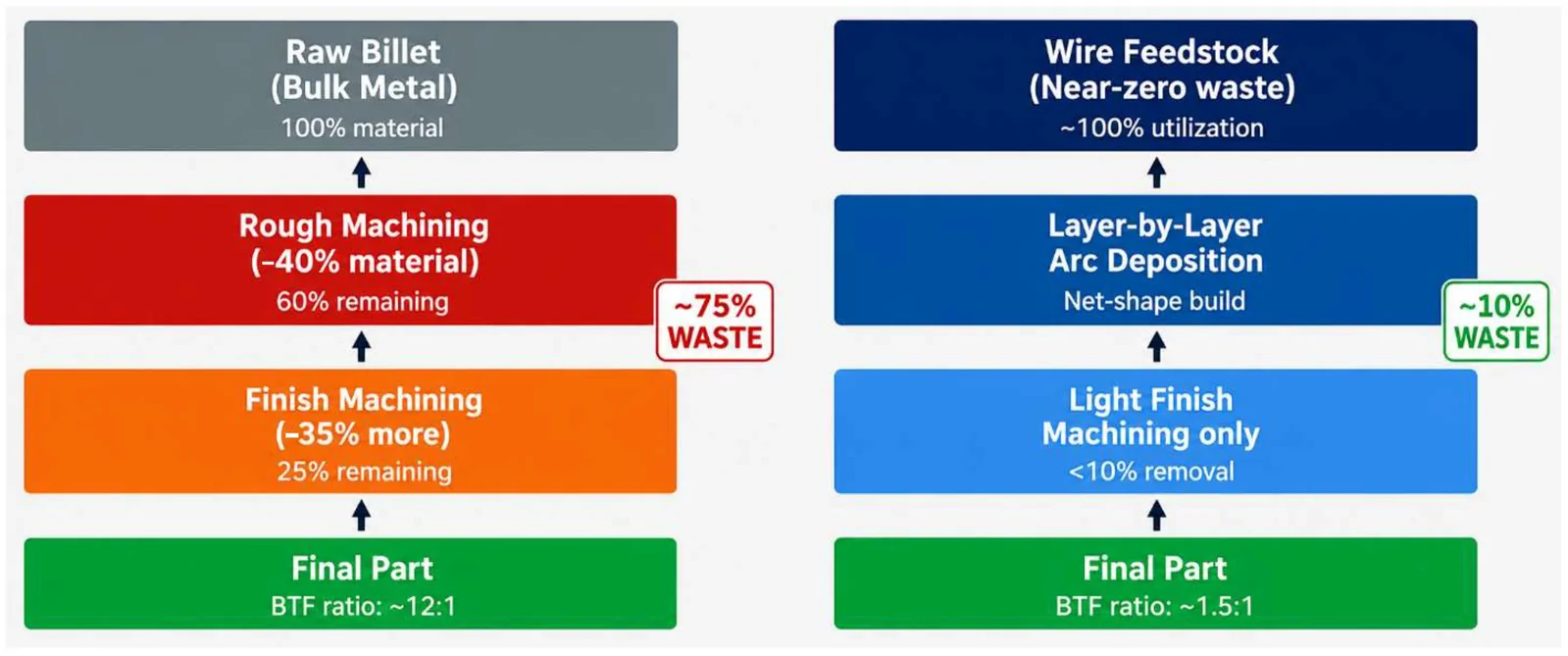
Comparison of traditional subtractive and WAAM additive manufacturing routes, illustrating the reduction in material waste and buy-to-fly (BTF) ratio achievable with WAAM. Conceptual schematic generated with ChatGPT (GPT-5) from author-specified content; material fractions and ratios are illustrative.

WAAM draws directly on the rich industrial heritage and established process science of GMAW (including its MIG and MAG variants), GTAW (TIG), and PAW processes, which have been refined over more than seven decades of industrial application. The conceptual roots of arc-based shape deposition trace to Baker’s 1925 patent on the deposition of successive weld beads to produce solid objects [8]. However, modern computer-controlled WAAM emerged in earnest during the 1990s and 2000s, with pioneering contributions from Cranfield University (the WAAM/WAAMMat program), aerospace research institutions, and other groups developing three-dimensional welding and hybrid welding–milling systems [9,10,11,12], targeting the fabrication of large titanium and high-strength steel aerospace structures with markedly reduced buy-to-fly ratios [13,14]. The comparison between traditional manufacturing and the WAAM route is shown in Figure 1.

Welding science and WAAM share a common metallurgical foundation, not merely the convenience of available equipment. From a physical metallurgy standpoint, WAAM and fusion welding share closely related governing phenomena: arc-driven metal melting, melt pool dynamics under electromagnetic, buoyancy, and Marangoni forces, solidification governed by local temperature gradient G and solidification rate R, solid-state phase transformations upon cooling, and the generation of residual stresses under constrained thermal conditions [15,16]. Yet WAAM imposes thermal conditions that differ from those of single-pass welding. Heat accumulation and repeated reheating of prior deposits also occur in multi-pass welding of thick sections, which may require hundreds of passes, so they are not unique to WAAM; what distinguishes WAAM is that the deposit is a free-standing structure that grows away from its only heat sink. The progressive increase in thermal resistance between the melt pool and the substrate, together with the evolving geometry of the build, produces microstructural heterogeneity, residual stress fields, and dimensional deviations that have no exact analog in conventional welding practice.

Although several reviews have addressed WAAM process development, alloy-specific properties, and defect mitigation [17,18], fewer studies have explicitly examined WAAM as a direct metallurgical extension of traditional arc welding processes. This review therefore emphasizes the transition from conventional welding to layer-wise additive manufacturing, focusing on how heat input, metal transfer, melt-pool solidification, repeated thermal cycling, and defect formation are altered when GMAW, GTAW, and PAW are adapted for WAAM.

The closest precedent is the review of Oliveira et al. [19], which revisited fundamental welding concepts for fusion-based additive manufacturing as a whole, including laser, electron-beam, and arc processes; identified the similarities in solidification, chemical reactions, thermal effects, residual stress, and distortion; and proposed geometric, energetic, and thermal criteria for process optimization together with a dimensionless energy-density parameter. The present review differs from it, and from recent general WAAM reviews [20,21], in three respects. First, it is restricted to arc-based wire deposition and treats the arc processes themselves, including metal transfer physics and the coupling between wire feed and heat input in GMAW and CMT, which is absent in GTAW and PAW. Second, it separates the thermal and solidification features that WAAM shares with multi-pass welding from those that are distinctive to WAAM and uses the *G*/*R* and *G*·*R* framework to state which microstructural outcomes arise from each. Third, it links these principles to quantitative data verified against the original publications, to hybrid deformation strategies, and to the barriers to industrial qualification.

Understanding these similarities and differences from first principles is the central intellectual challenge of WAAM process science and the primary motivation for this review. The objective of this review is threefold: (i) to provide a comparative analysis of GMAW, GTAW, and PAW in the context of WAAM applicability; (ii) to critically examine the physical mechanisms of melt pool formation, solidification, phase transformation, and defect generation in WAAM relative to traditional welding, supported by governing equations; and (iii) to synthesize the current understanding of microstructural evolution and defect formation across alloy systems, while identifying open research challenges and future directions. The review is structured to serve both researchers entering the field and practitioners seeking to optimize WAAM for industrial deployment.

## 2. Scope and Literature Selection

**Figure 2:**
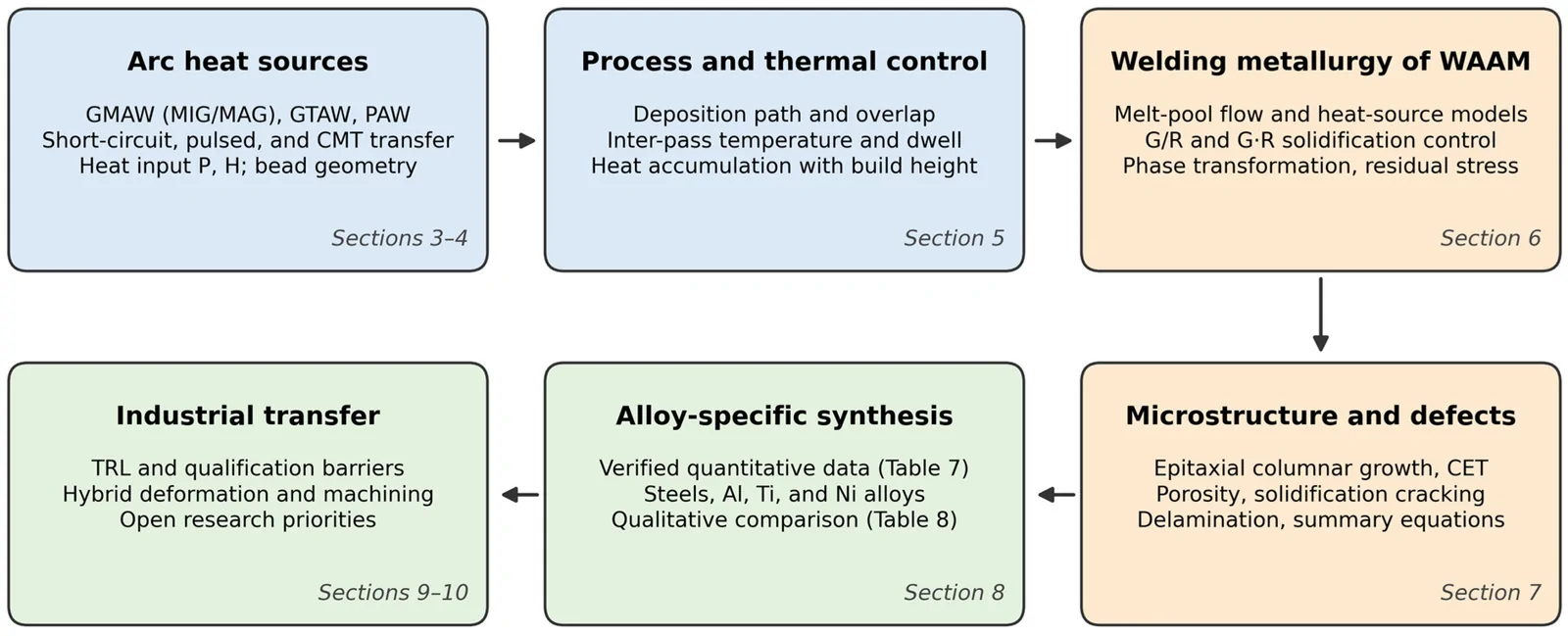
Scope and organization of this review, linking the arc heat sources and process control (Section 3, Section 4 and Section 5) to welding metallurgy, microstructure, and defects (Section 6 and Section 7), alloy-specific synthesis (Section 8), and industrial transfer and hybrid strategies (Section 9 and Section 10). Schematic drawn with Python 3.12 with Matplotlib 3.10; no data are shown.

This article is a critical narrative review. It is not a systematic review: the literature was selected for its relevance to the process–structure–property questions addressed here, not through a protocol-driven search with documented record counts, and no meta-analysis was performed. The scope and organization of the review are summarized in Figure 2.

### 2.1. Literature Identification and Selection Principles

The review addresses the literature published between 2005 and March 2025 on arc-based wire additive manufacturing, together with earlier foundational works on welding metallurgy, solidification, and three-dimensional welding (from 1925 onward) where these are needed to explain the governing physics; during the revision of this article in 2026, the literature base was updated with publications from 2025 and 2026, including recent reviews and studies on WAAM feedstock, process configuration, and hybrid deformation processing. The searches combined three concept areas: (i) the process (wire arc additive manufacturing); (ii) the arc heat source (GMAW, including MIG and MAG; GTAW; PAW; and CMT); and (iii) process–structure outcomes (heat input, deposition path, solidification, microstructure, porosity, and residual stress). The literature was identified mainly through Google Scholar searches, and full texts were obtained through the library resources of Wayne State University. Further references were provided by the co-authors through the library resources of their institutions. Conceptual schematic illustrations were generated with ChatGPT (GPT-5, OpenAI, San Francisco, CA, USA) from content specified by the authors and were reviewed and revised by the authors; the plotted figures and the graphical abstract were drawn with Python 3.12 (Python Software Foundation, Wilmington, DE, USA) and Matplotlib 3.10 code prepared with the assistance of Claude (Anthropic, San Francisco, CA, USA). The method used is stated in the caption of each figure. None of these figures presents measured, digitized, or extracted data; the inter-pass cooling curves in Section 5.2 are calculated from Equation (14).

Studies were selected according to the following principles: (i) peer-reviewed journal articles and conference papers in English form the primary evidence base, and theses are cited only where the corresponding data have not been published in journal form; (ii) experimental, computational, and combined studies were considered if they address GMAW-, GTAW-, or PAW-based wire deposition of steels, aluminum alloys, titanium alloys, nickel-based superalloys, or copper alloys; (iii) review articles, textbooks, standards, and patents are cited for context and background but are not used as sources of quantitative data; and (iv) studies reporting only qualitative observations are used for mechanistic discussion, whereas quantitative values are taken only from studies that meet the criteria in Section 2.3.

Because the literature was identified through relevance-ranked Google Scholar searches, whose ranking and coverage are not fully transparent, and because the search dates and record counts were not documented, this review does not claim complete or reproducible coverage of the WAAM literature. To limit selection bias in the quantitative synthesis, every numerical value reported in the table of verified quantitative data in Section 8.1 was verified against the original publication, and figures that show trends without numerical data are identified as conceptual schematics.

### 2.2. Data Extraction and Quality Assessment

For each study considered for the quantitative comparison, the following information was recorded where reported: alloy system and composition, WAAM process variant (GMAW, GTAW, PAW, CMT), linear heat input H (J/mm), travel speed, wire feed rate, inter-pass temperature, resulting grain size (µm), porosity fraction (%), Vickers hardness (HV), and ultimate tensile strength (MPa). Studies that reported only qualitative microstructural observations without quantitative data were retained for mechanistic discussion but excluded from the quantitative comparison in Section 8.1. The quality of each study was screened against three author-defined criteria: physical consistency of reported data, sample size and statistical treatment, and independent validation of key claims.

The WAAM literature suffers from important methodological heterogeneity: significant variations in reporting standards, specimen geometry, measurement locations within builds, and testing protocols make direct quantitative comparisons challenging. Accordingly, quantitative values extracted from the literature were used for representative comparison rather than direct statistical normalization across studies. The consequences of this limitation for the quantitative synthesis are addressed in Section 8.1 and considered further in Section 10.1.

### 2.3. Criteria for the Quantitative Comparison

A study was used as a data source for the quantitative comparison in Section 8.1 only if it (i) was a primary experimental study rather than a review, textbook, or schematic source; (ii) identified the alloy and the arc-process variant; (iii) reported the quantity numerically in its text or tables together with the process condition to which the value refers; and (iv) was available in full text for verification. Values were transcribed as reported; no values were digitized from plots, interpolated, or converted other than as stated in the table. Where a study reported several conditions, the controlled variable and the condition supporting each value are identified in the table. Figures in this review that show trends without numerical data are identified in their captions as author-drawn schematics.

## 3. WAAM Process Architecture and Comparison with Laser-Based AM

### 3.1. WAAM System Architecture and Governing Heat Input

**Figure 3:**
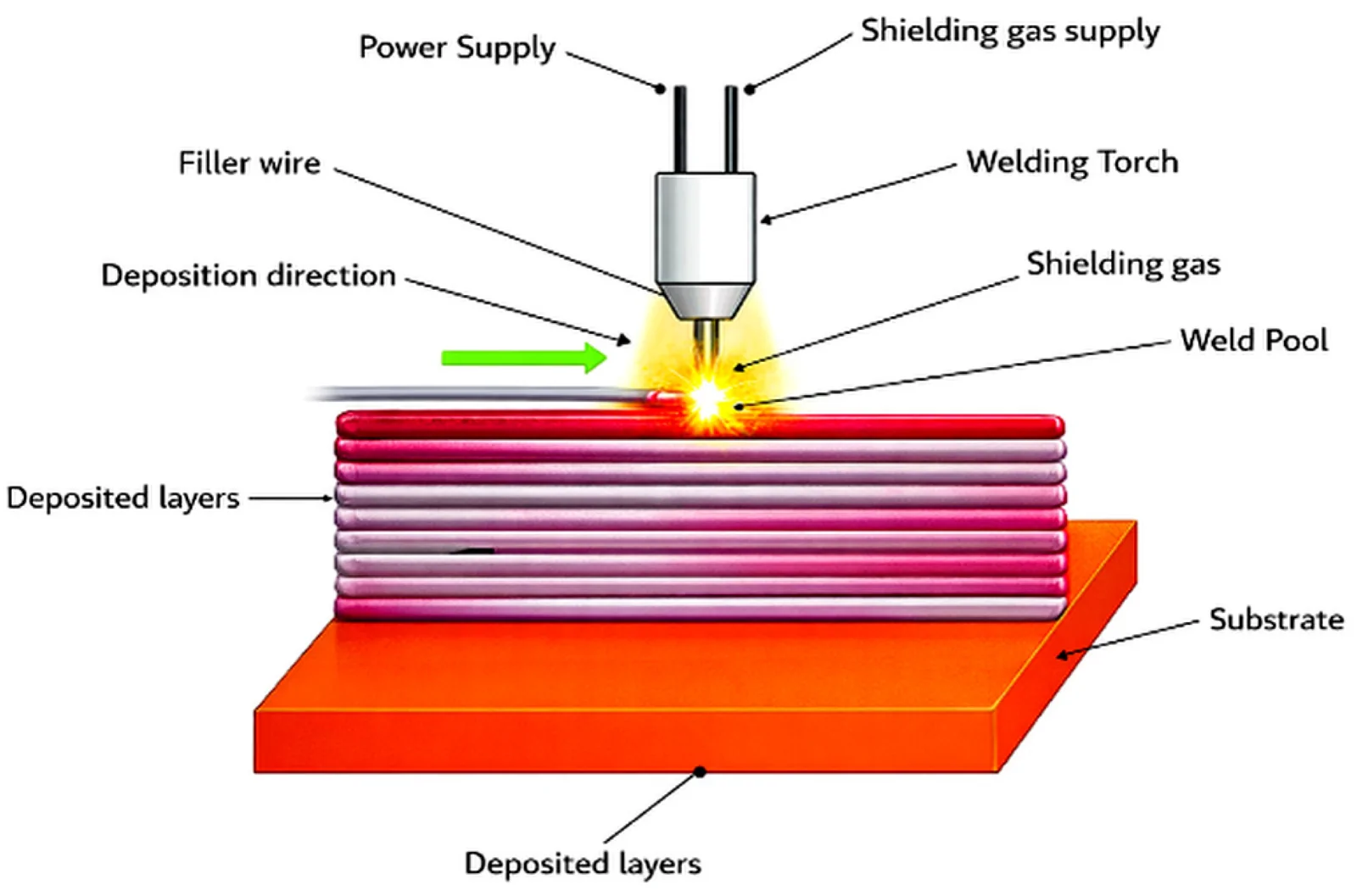
Schematic of the WAAM layer-by-layer deposition process showing the welding torch, power supply, shielding gas nozzle, filler wire feed, weld pool, and the growing multi-layer deposit structure on the substrate. The deposition direction and layered build geometry are indicated. Conceptual schematic generated with ChatGPT (GPT-5) from author-specified content.

**Figure 4:**
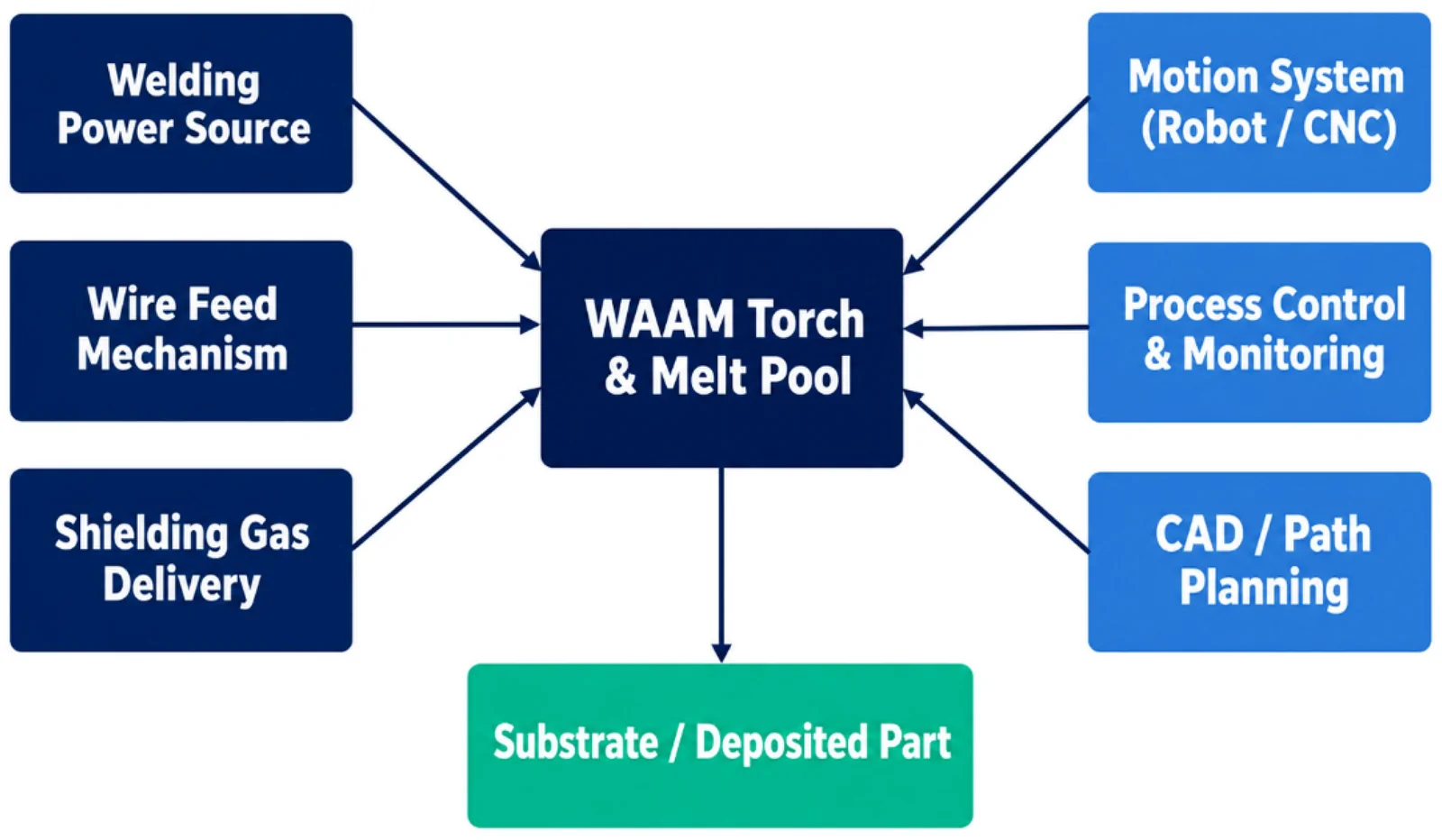
Schematic of a WAAM system showing the five principal sub-systems: welding power source, wire feed mechanism, motion system, shielding gas delivery, and process control. Conceptal schematic generated with ChatGPT (GPT-5) from author-specified content.

A WAAM system comprises five principal sub-systems [22], as shown schematically in Figure 3 and Figure 4: (i) a welding power source, such as a constant-voltage GMAW inverter, GTAW unit, or plasma power supply; (ii) a wire feeding mechanism delivering feedstock at controlled rates; (iii) a motion system typically a 6-axis industrial robot or CNC gantry that controls torch position, travel speed and deposition trajectory; (iv) a shielding gas delivery system; and (v) a process control and monitoring system coordinating all sub-systems [23]. The quality and geometric accuracy of WAAM parts are governed by a complex interplay of process parameters, physical phenomena, and geometrical features, as classified by Jafari et al. [24]. The importance of this system architecture in WAAM is that each sub-system influences not only bead formation, as in conventional welding, but also the accumulated thermal history and dimensional stability of the entire build.

Tooling and support structures play different roles in welding and in WAAM. In welding, fixtures and clamps locate the parent members, control fit-up and distortion, and backing bars or strips support the root, while the parent plates themselves provide most of the heat sink. In WAAM, the substrate is the only initial support and heat sink, and it becomes part of the thermal and mechanical boundary condition of the build: its thickness, clamping, and preheat set the early-layer cooling rate, and the distortion that develops during deposition is partly released when the part is unclamped [25,26]. As the deposit grows, its stiffness, heat-extraction path, and exposed surface area change continuously, so the thermal boundary conditions evolve during manufacturing instead of remaining fixed as they do for a joint. Active cooling of the substrate has therefore been used to control heat accumulation in WAAM [27], and overhanging or inclined features are usually produced by torch inclination, positioner rotation, or deposition sequence rather than by sacrificial support structures [21].

The deposition process is described at the most fundamental level by the net heat input per unit length, which represents the primary process-level control variable linking power source settings to thermal conditions in the deposit. The effective arc power *P* (W) and the net linear heat input *H* (J/mm) are given by:

(1) $$P=\eta VI,\;\;H=\frac{P}{v}=\frac{\eta VI}{v}$$

where *η* is the arc thermal efficiency (dimensionless; typically about 0.80–0.90 for GMAW, 0.65–0.75 for GTAW, and 0.5–0.75 for PAW, in which part of the arc energy is lost to the water-cooled constricting nozzle [16]), *V* is arc voltage (V), *I* is welding current (A), and *v* is travel speed (mm/s). *P* is the heat-source power that enters point-source, line-source, and volumetric heat-flow models (Equations (2), (3), (15) and (16)), whereas *H* is the energy delivered per unit length of deposit and is used to compare process conditions; the two quantities have different units and must not be interchanged. *H* influences the peak melt-pool temperature, the depth of fusion into the prior layer, the cooling rate, and the resulting microstructure [16].

The quasi-steady temperature field around a moving arc can be approximated by the analytical solutions of Rosenthal [28], whose form depends on the assumed heat-flow geometry. For a point source moving over a thick (semi-infinite) substrate, heat flow is three-dimensional and the solution is Equation (2); for a line source moving through a thin plate of thickness d, heat flow is two-dimensional and the solution is Equation (3):

(2) $$T-T_{0}=\frac{P}{2\pi \lambda s}exp\left[-\frac{v\left(s+\xi \right)}{2\alpha }\right]$$

(3) $$T-T_{0}=\frac{P}{2\pi \lambda d}exp\left(-\frac{v\xi }{2\alpha }\right)K_{0}\left(\frac{vr}{2\alpha }\right)$$

where *T*_0_ is the preheat or inter-pass temperature (K), *P* is the effective arc power (W) from Equation (1), *λ* is the thermal conductivity (W·m^−1^·K^−1^), *ρ* is the density (kg·m^−3^), *c* is the specific heat (J·kg^−1^·K^−1^), *α* = *λ*/(*ρc*) is the thermal diffusivity (m^2^·s^−1^), *v* is the travel speed (m·s^−1^), *ξ* = *x* − *vt* is the coordinate along the travel direction in the frame moving with the source, d is the plate or wall thickness (m), *s* = √(*ξ*^2^ + *y*^2^ + *z*^2^) and r = √(*ξ*^2^ + *y*^2^) are the radial distances from the source, and *K*_0_ is the modified Bessel function of the second kind of order zero. In both expressions the prefactor has the units W/(W·m^−1^·K^−1^·m) = K. The first layers deposited on a thick substrate are closer to the three-dimensional case, whereas heat flow in a thin wall several bead widths high is closer to the two-dimensional case with d equal to the wall thickness. Because both solutions assume constant properties, a concentrated source, and no latent heat or melt-pool convection, they do not capture the full complexity of multilayer WAAM; they nevertheless provide a first-order analytical framework for understanding how process parameters control the isotherms that define melt-pool geometry [29].

### 3.2. Comparison of WAAM with Laser-Based Additive Manufacturing

WAAM is most frequently contrasted with LPBF and Laser DED. While laser-based processes excel in geometric resolution and surface finish, WAAM offers clear advantages in deposition rate, component scale, material utilization and economic accessibility. Table 1 presents a process-level comparison based on the work of Ding (2012) [30] and subsequent contributions from the literature.

As shown in Table 1, WAAM’s combination of high energy efficiency (>80%), high deposition rate (up to 10 kg/h), near-100% material utilization, and large-scale build capability positions it as a strong candidate for structural applications where gross material removal and complex internal features are not required. The compromise in surface finish and maximum component complexity compared to powder-bed processes is typically addressed through hybrid additive–subtractive machining, which has emerged as WAAM’s natural complement in industrial practice [9,10].

A commonly overlooked comparison metric is the buy-to-fly (BTF) ratio, defined as the mass of raw material consumed per unit mass of finished component. For machined aerospace titanium components, BTF ratios of 12:1 to 20:1 are common, whereas WAAM can reduce this to 1.5:1 to 3:1 for equivalent geometries, representing substantial material cost savings and corresponding reductions in machining time and energy consumption [1,14].

## 4. Arc Welding Processes and Metal Transfer Modes in WAAM

Having established the basic WAAM system architecture and heat-input framework, the next step is to examine how conventional arc welding processes operate when they are adapted for layer-wise additive deposition. GMAW, GTAW, and PAW provide the principal arc-based heat sources for WAAM, but they differ markedly in heat input, deposition rate, arc stability, shielding control, equipment complexity and alloy suitability [35].

### 4.1. GMAW, GTAW and PAW as WAAM Heat Sources

Gas Metal Arc Welding (GMAW) is the most widely employed heat source in WAAM. GMAW denotes the process category: it is designated metal inert gas (MIG) welding when the shielding gas is inert (Ar, He, or Ar–He mixtures, as used for aluminum, copper, and nickel alloys) and metal active gas (MAG) welding when the shielding gas contains an active component such as CO_2_ or O_2_ (e.g., Ar–CO_2_ mixtures for carbon and low-alloy steels) [16,36]. The general term GMAW is used in this review unless a cited study specifically employed MIG or MAG conditions. Commercially introduced in the late 1940s, GMAW establishes an arc between a continuously fed consumable wire electrode and the workpiece, which serves as the substrate or previously deposited layer in AM terminology, melting both to form the melt pool. Shielding is provided coaxially through the torch nozzle using argon, helium, or Ar–CO_2_ mixtures [36]. Key process parameters in GMAW-WAAM are arc voltage V, welding current I (or wire feed rate WFR, which determines I in constant-voltage mode), travel speed v, wire stick-out distance L, wire diameter d, and shielding gas composition X. The relationship between WFR and the resulting bead dimensions has been studied extensively [37,38,39].

Dickens et al. [40] conducted parameter studies on mild steel WAAM using Fe–C (0.08%)–Si (0.9%)–Mn (1.5%) wire and documented the influence of voltage, WFR, stick-out, wire diameter, and travel speed on arc current, bead width W_b_, and bead height H_b_. Response surface methodology (RSM) and analysis of variance (ANOVA) are commonly used to rank the influence of these parameters on bead width and height, and empirical second-order regression models of the following form are widely applied [37,38,39]:

(4) $$W_{b}=a_{0}+a_{1}V+a_{2}I+a_{3}v+a_{4}VI+a_{5}Iv+a_{6}Vv+a_{7}V^{2}+a_{8}I^{2}+a_{9}v^{2}$$

(5) $$H_{b}=b_{0}+b_{1}V+b_{2}I+b_{3}v+b_{4}VI+b_{5}Iv+b_{6}Vv+b_{7}V^{2}+b_{8}I^{2}+b_{9}v^{2}$$

where *V* is arc voltage (V), *I* is welding current (A), *v* is travel speed (mm·s^−1^), *W_b_* is bead width (mm), *H_b_* is bead height (mm), and *a_i_*, *b_i_* are empirically determined regression coefficients. The three quadratic terms (*V*^2^, *I*^2^, *v*^2^) and three two-way interaction terms (*V*·*I*, *I*·*v*, *V*·*v*) form a full second-order response surface model, consistent with the RSM framework applied in [37,38,39]. The sign and relative magnitude of the coefficients in Equations (4) and (5) encode the parametric hierarchy: a_1_ and a_2_ are typically large and positive for W_b_, while b_3_ is negative (higher travel speed reduces H_b_ at fixed arc power), reflecting the process physics discussed in Section 3.1. These parametric relationships are essential for WAAM process planning because *W_b_* and *H_b_* together determine the layer overlap ratio, step-over distance *d_so_*, and overall surface waviness of the deposited structure [38,39].

The industrial appeal of GMAW for WAAM rests on broad alloy compatibility, suitability for multiple welding positions, high productivity, ready automation, and considerably lower capital and operating costs than laser or electron beam systems. The coupling of a GMAW torch to a CNC milling machine, creating a hybrid additive–subtractive system, has been demonstrated by multiple research groups and represents one of the most economically accessible routes to metallic AM for small and medium enterprises [11,41].

**Figure 5:**
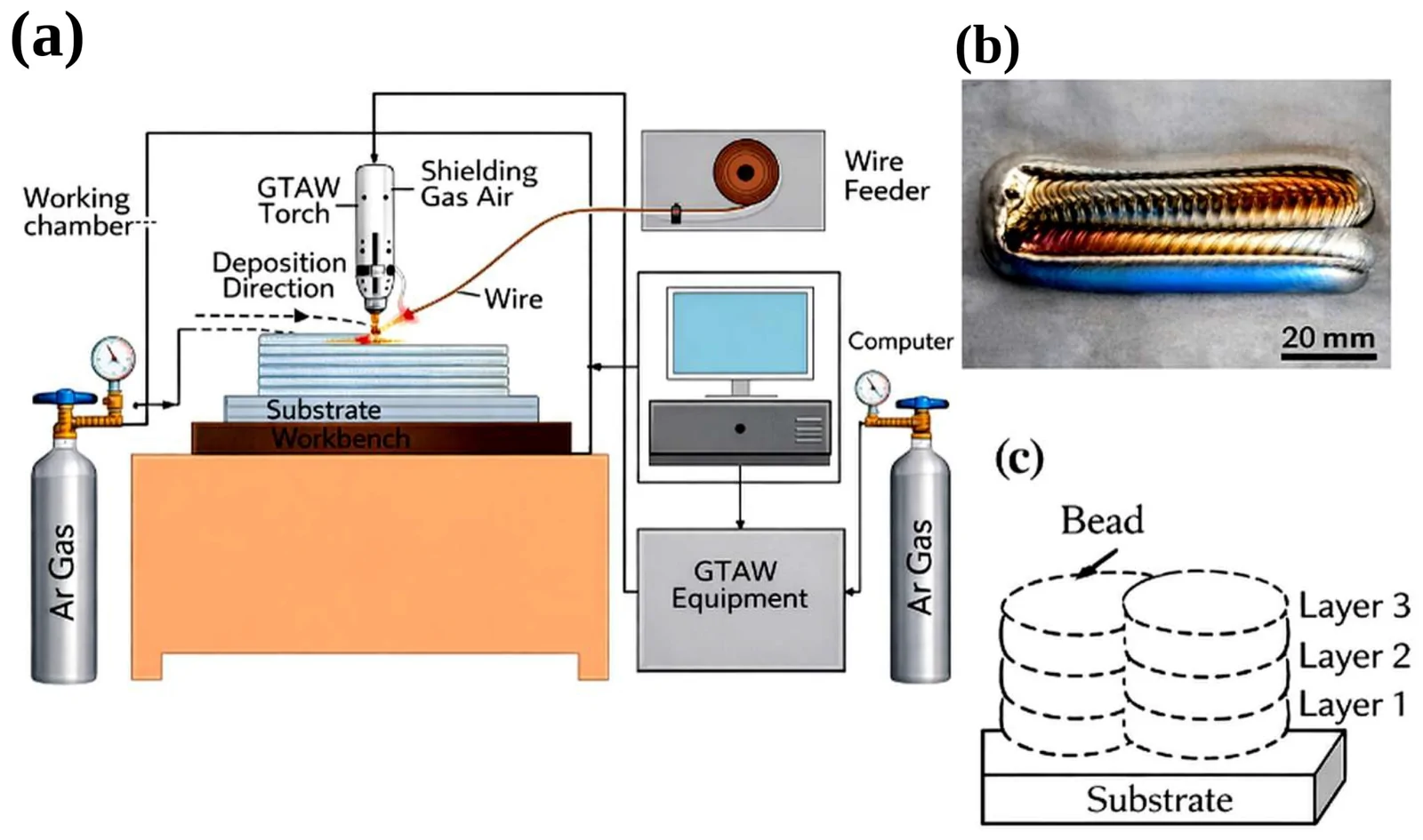
GTAW-based WAAM experimental system: (**a**) schematic of the working chamber showing GTAW torch, shielding gas inlets, wire feeder, substrate, and welding power supply; (**b**) photograph of a representative multi-pass deposited wall; (**c**) schematic cross-section showing individual bead geometry and layer stacking on the substrate. Schematics (**a**,**c**): authors’ own work. Photograph (**b**): original, courtesy of the authors.

Gas Tungsten Arc Welding (GTAW), or TIG welding, employs a non-consumable tungsten electrode to generate the arc and delivers filler wire separately into the melt pool, as shown in Figure 5. In WAAM, GTAW is employed where deposit quality, arc stability, and shielding control are prioritized over deposition rate, particularly for reactive metals such as titanium and nickel-base superalloys where oxygen pickup must be minimized [32].

Porosity is the principal quality concern in aluminum WAAM [42,43], and the hot-wire GTAW variant (HWAAM) has been investigated as a way to increase deposition rate while reducing it. Fu et al. [44] showed that in hot-wire arc additive manufacturing of an aluminum alloy the pores clustered at the interlayers and the porosity passed through a minimum at a hot-wire current of 100 A, at which the relative density increased from 96.8% without hot-wire current to 99.6%; at higher hot-wire currents the porosity increased again, illustrating the trade-off between productivity and deposit quality. Because the linear heat input controls melt-pool size and cooling rate, the grain size $\bar{d}$ is often correlated empirically with *H*:

(6) $$\bar{d}\propto H^{m}$$

where m is an empirical exponent that depends on the alloy, the process variant, and the heat-input regime; it must be fitted to data for a given process window and is not a universal constant.

Plasma Arc Welding (PAW) constricts the arc through a fine orifice in the nozzle, producing a highly concentrated plasma jet with energy densities higher than those typically obtained in GMAW or GTAW arcs. The constriction increases the power density and produces a deeper and narrower melt pool than an unconstricted arc at the same power [16]. The resulting solidification conditions (*G*, *R*, and the cooling rate *G*·*R*) depend on the travel speed and deposit geometry and must be evaluated for the specific process window rather than inferred from arc constriction alone [32]. In WAAM, PAW is preferred for applications where high travel speed, high-quality deposits, and reduced distortion are simultaneously required.

### 4.2. Comparative Assessment of Arc-Based WAAM Processes

**Figure 6:**
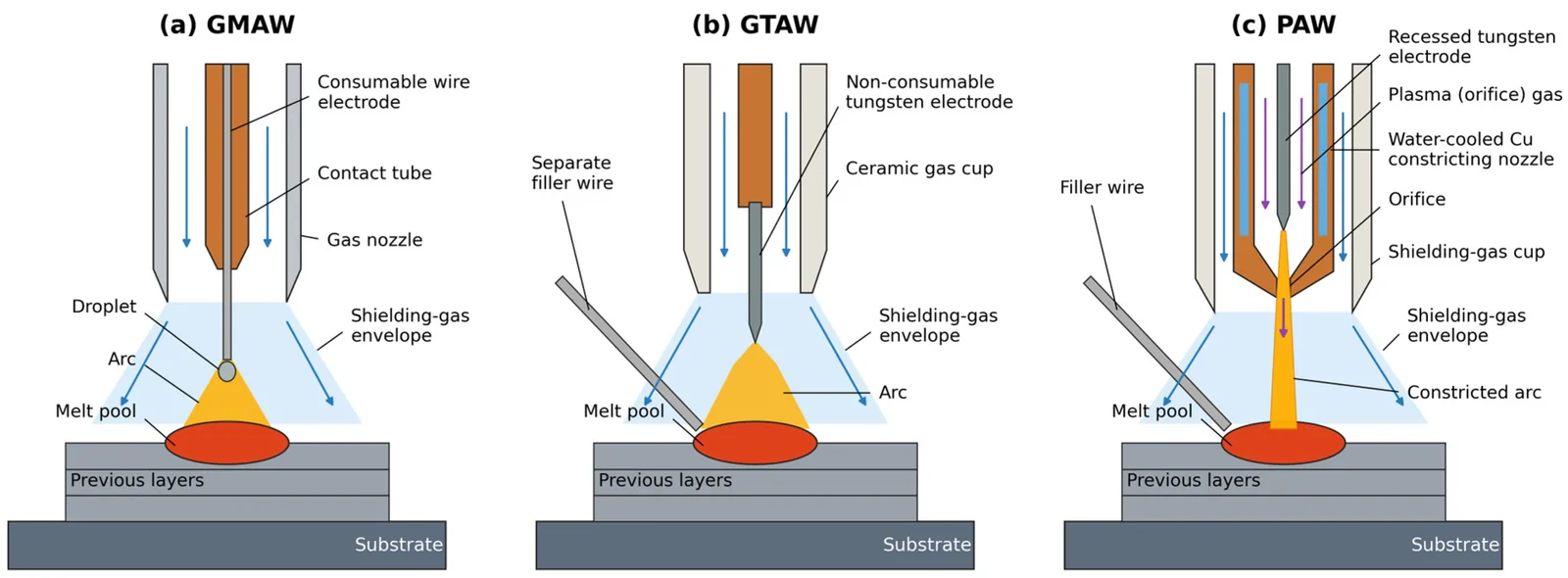
Schematic comparison of the three arc processes used in WAAM: (**a**) GMAW, with a consumable wire electrode fed through the contact tube and a coaxial shielding-gas flow; (**b**) GTAW, with a non-consumable tungsten electrode, a ceramic gas cup, and a separately fed filler wire; (**c**) PAW, with the tungsten electrode recessed inside a water-cooled copper constricting nozzle, plasma (orifice) gas flowing around the electrode and through the orifice, and a separate outer shielding-gas flow. In all panels, the shielding gas is shown as an envelope surrounding the arc and melt pool. Drawn with Python (Matplotlib 3.10).

The trade-off for PAW’s superior arc characteristics is considerably higher equipment cost. This differential has, to date, limited PAW-WAAM primarily to high-value aerospace and defense applications. Table 2 and Figure 6 summarize the comparative suitability of GMAW, GTAW, and PAW for WAAM application selection. Therefore, the selection of GMAW, GTAW, or PAW for WAAM should not be based only on deposition rate. It should also consider thermal input, shielding quality, melt-pool stability, dimensional accuracy, and alloy sensitivity to oxidation or cracking. Among these processes, GMAW requires additional attention because its thermal behavior and bead stability are strongly governed by metal transfer mode.

A further difference that governs process selection is whether material feed and heat input can be set independently. In GMAW and CMT, the wire is the electrode: in constant-voltage operation the wire feed speed sets the current, so the deposition rate, the arc power, and therefore the linear heat input H are coupled, and commercial synergic programs further tie voltage and waveform to a single wire-feed setting. Deposition rate, bead geometry, and thermal conditions therefore cannot always be optimized separately when productivity, geometric accuracy, and thermal requirements conflict. In GTAW and PAW, the filler wire is fed independently of the arc current, so the energy delivered per unit mass of deposited metal can be varied at a fixed deposition rate. In PAW-based deposition, wire feed speed, arc current, and plasma gas flow rate each influence droplet transfer and bead formation [45], and hot-wire feeding adds resistive preheating of the wire that raises the deposition rate without increasing the arc power [44]. This flexibility is one reason why GTAW and PAW are used for titanium alloys [13,46], whereas the coupled but more productive GMAW and CMT processes dominate the deposition of steels and aluminum alloys.

### 4.3. Metal Transfer Physics in GMAW-WAAM

In GMAW, molten metal is transferred from the consumable wire electrode to the melt pool by mechanisms governed by the competition among arc pressure, electromagnetic (Lorentz) force, surface tension, and gravity. The detachment of a droplet occurs when the sum of detaching forces exceeds the retaining surface tension force. The dominant retaining force for a droplet of radius r_d_ hanging from a wire of radius r_w_ at its tip is the surface tension force *F_γ_*:

(7) $$F_{\gamma }=2\pi r_{w}\gamma_{LV}$$

where *γ_LV_* is the liquid–vapor surface tension (N/m). For a pendant droplet with the arc attached over its lower surface, the electromagnetic (Lorentz) force acting to detach the droplet can be expressed as [47]:

(8) $$F_{em}=\frac{\mu_{0}I^{2}}{4\pi }\left[ln\frac{r_{d}sin\theta }{r_{w}}-\frac{1}{4}-ln\frac{1}{1-cos\theta }+\frac{2}{{\left(1-cos\theta \right)}^{2}}ln\frac{2}{1+cos\theta }\right]$$

where *μ*_0_ = 4π × 10^−7^ H/m is the magnetic permeability of free space, *r_d_* is the droplet radius, and *θ* is the angle that defines the extent of the arc-attachment (current-conducting) region on the droplet surface. As current *I* increases, *F_em_* increases approximately with *I*^2^, eventually overcoming *F_γ_* and enabling finer, more frequent droplet detachment which is associated with the transition from globular to spray arc transfer [47]. This transition current I_tr_ depends on wire composition and diameter *d_w_* approximately as:

(9) $$I_{tr}\approx C_{1}d_{w}^{\;n}{\left(\rho_{w}\gamma_{LV}\right)}^{1/2}$$

where *C*_1_ and n are empirical constants fitted for a given wire–gas combination [47]; Equation (9) is a scaling relation rather than a predictive model. For 1.1–1.2 mm carbon-steel wire in argon-rich shielding gas the transition current is typically about 220–240 A, whereas for 1.2 mm aluminum wire it is about 130–135 A [36], which explains why spray arc transfer in aluminum occurs at considerably lower currents than in steel.

**Figure 7:**
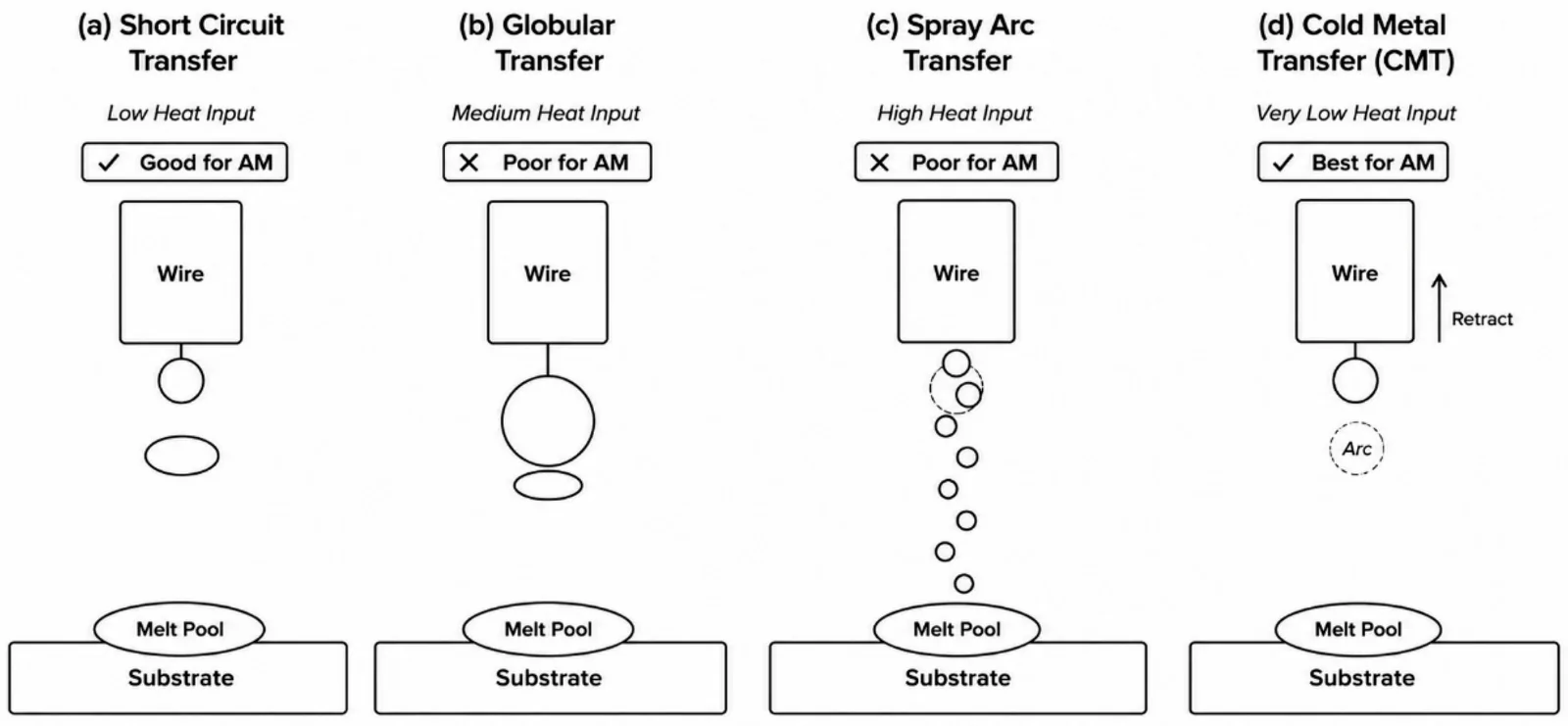
Schematic illustration of the four GMAW metal transfer modes: (**a**) Short Circuit, (**b**) Globular, (**c**) Spray Arc, and (**d**) Cold Metal Transfer (CMT), with their respective heat input levels and AM suitability. Conceptual schematic generated with ChatGPT (GPT-5) from author-specified content.

The key point for WAAM is that the heat input requirement for additive deposition differs from that for conventional fusion welding. In traditional fusion welding, high heat input is required to achieve full penetration and complete fusion of base plates. In WAAM, the objective is controlled fusion with the previously deposited layer, usually through shallow remelting sufficient to achieve metallurgical bonding rather than full plate penetration. This requirement favors operation in a lower heat input regime than many conventional welding applications, shifting the optimal GMAW operating mode toward short-circuit transfer and, where available, Cold Metal Transfer. Table 3 and Figure 7 summarize the principal GMAW metal transfer modes and their implications for WAAM heat input, bead regularity, spatter, and process stability.

### 4.4. Short-Circuit Transfer and Cold Metal Transfer

In Short Circuit Transfer (SCT), the energized wire periodically contacts the melt pool at 90–200 Hz. At each contact, the arc extinguishes and a short-circuit current flows. The electromagnetic pinch force severs the wire to deposit a small droplet; the arc re-ignites and the cycle repeats. The per-cycle energy *E_cyc_* deposited is approximately:

(10) $$E_{cyc}=\int_{0}^{t_{arc}}V_{arc}I_{arc}\;dt+\int_{0}^{t_{sc}}V_{sc}I_{sc}\;dt$$

where *t_arc_* and *t_sc_* are arc-on and short-circuit durations respectively. SCT’s low average current and voltage, and therefore low H, align well with WAAM’s requirement for controlled inter-layer remelting. Its principal drawbacks are limited deposition rate and moderate spatter, which can contribute to surface roughness, bead irregularity, and material waste [49]. Waveform-controlled short-circuiting processes can relax the deposition-rate limitation: Panchenko et al. [50] reported deposition rates of up to 2.2 kg/h for Al–Mg–Mn walls with a heat input 16% lower than that of a conventional self-regulated GMAW process.

Cold Metal Transfer (CMT), developed by Fronius International, is among the most thermally efficient GMAW modes currently used for WAAM. In CMT, the wire feed motion and welding current are electronically synchronized: as the wire approaches the melt pool and makes contact, the current is reduced to near zero and the wire is retracted to mechanically assist droplet detachment at up to 70 Hz. This controlled retraction eliminates the uncontrolled arc-off phase associated with conventional SCT, thereby lowering heat input, spatter, and distortion in many GMAW-WAAM applications [49].

Galeazzi et al. [51] compared conventional GMAW with the CMT, CMT Advanced, and CMT Pulse variants for thin walls of AISI 420 martensitic stainless steel and found that CMT Advanced and CMT Pulse lay at opposite extremes of the tested range: CMT Advanced gave the lowest heat input and the best wall-height performance, whereas CMT Pulse lay at the high-heat-input end. The metallurgical consequence of such differences can be interpreted through the inverse dependence of grain size, or of the scale of the transformation products, on the cooling rate:

(11) $$\bar{d}\propto {\left(\frac{dT}{dt}\right)}^{-n}$$

where *n* is an empirical, alloy-dependent exponent.

A higher cooling rate shortens the time available for grain-boundary migration and for coarsening of transformation products. A variant that raises the energy delivered per unit length, such as CMT Pulse, is therefore expected to enlarge the melt pool, extend the time spent at high temperature, and lower the cooling rate through the relevant temperature range, all of which favor coarser structures; conversely, a low-heat-input variant such as CMT Advanced is expected to favor finer structures. Grain size must therefore be interpreted together with the heat input per cycle and the reheating history, and a larger grain size should not be associated with a higher cooling rate unless an additional mechanism, such as reheating by subsequent layers, is demonstrated. This interpretation is consistent with infrared measurements on ER309LSi thick walls by Silva et al. [52]: at the same wire feed rate, CMT Pulse delivered a higher power than CMT; without interlayer idle time, the area above 900 °C in the last layer was 1013 mm^2^ for CMT Pulse and 400.8 mm^2^ for CMT, and the mean interlayer temperature was on average 15.0% higher for CMT Pulse; interlayer idle times of up to 300 s controlled heat accumulation for both variants. Separately, Luo et al. [53] compared pulsed and non-pulsed arcs in WAAM of aluminum alloys and found that the pulsed arc gave a higher droplet-transfer frequency, smaller droplets, and a higher deposition efficiency at similar arc power; because the arc force on the droplet also increased with arc power and could cause collapse of the deposited layer, they recommended the lowest pulsed arc power compatible with the required productivity.

Despite its thermal advantages in many WAAM applications [54], CMT adoption remains constrained by higher equipment cost, approximately two to three times that of conventional GMAW, and greater system complexity. As CMT technology matures and competition reduces costs, its adoption in WAAM is expected to increase considerably. Metal-transfer-mode selection in GMAW-WAAM is therefore a thermal and geometric control strategy rather than only a droplet detachment mechanism, because transfer stability directly affects heat input, remelting depth, bead regularity, spatter, and defect formation.

## 5. Deposition Path Planning and Thermal Management in WAAM

Beyond heat-source selection, WAAM build quality is strongly affected by the path followed by the torch and by the thermal state retained between successive layers. Deposition path strategy determines not only geometric fidelity, but also local reheating, cooling intervals, residual stress distribution and microstructural uniformity. For this reason, path planning and inter-pass temperature control should be considered together as coupled thermal-management strategies.

### 5.1. Path Planning Fundamentals and Deposition Patterns

Deposition path planning is among the most consequential decisions in WAAM process design. The path determines not only geometric fidelity, but also the thermal history experienced at each material location, which in turn governs microstructure, residual stress distribution, and mechanical properties. Jafari et al. [24] identified deposition path strategy as a primary determinant of WAAM microstructure. Ding et al. [55] demonstrated that, for thin-walled structures, optimal step-over distance d_SO_ can more than double material efficiency relative to an unoptimized baseline. The step-over distance governs the overlap ratio ω:

(12) $$\omega =1-\frac{d_{SO}}{W_{b}}$$

where *W_b_* is the single-bead width. For a parabolic single-bead profile, Ding et al. [56] showed with a tangent overlapping model that stable multi-bead overlapping requires a center distance of at least approximately 0.738 *W_b_* (*ω* ≈ 0.26); the conventional flat-top overlapping model gives 0.667 *W_b_* (*ω* ≈ 0.33), which the tangent model predicts to be unstable. A smaller overlap than this leaves valleys between beads, whereas a larger overlap causes material build-up and an unstable overlapping process.

**Figure 8:**
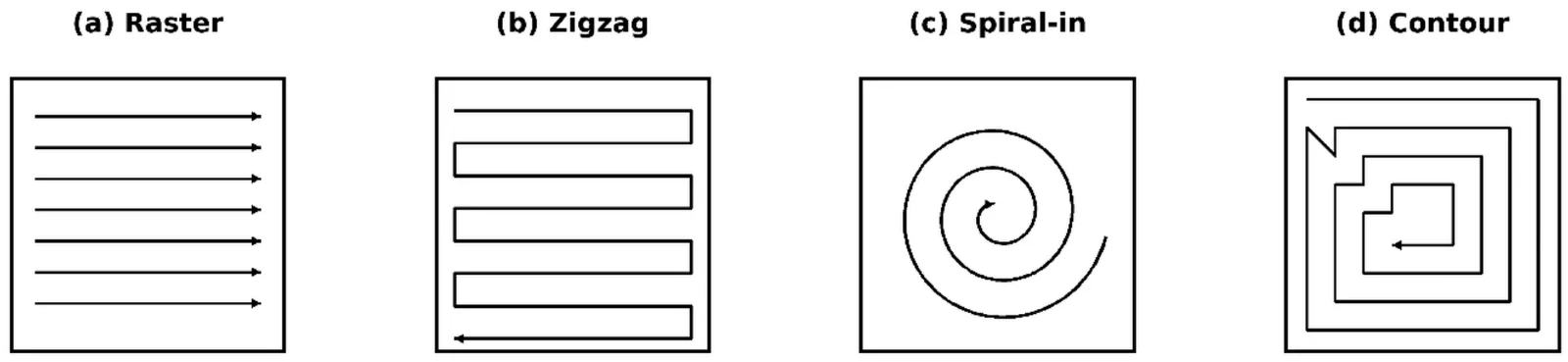
Classical WAAM deposition path patterns: (**a**) Raster parallel bidirectional lines; (**b**) zigzag continuous single bidirectional pass; (**c**) spiral-in inward continuous spiral; (**d**) contour successive boundary offsets.

Four classical deposition path patterns are used in WAAM, each with distinct thermal, geometric, and efficiency characteristics, as shown in Figure 8.

- Raster: Parallel lines are deposited in alternating directions. Its advantages include reduced heat accumulation due to cooling intervals between adjacent lines. Its limitations include frequent arc starts and stops, which may introduce bead-height variability, and boundary discretization errors that reduce geometric accuracy at perimeter regions [57].
- Zigzag: Parallel lines are connected into a single continuous bidirectional pass. Its advantages include fewer arc-start and arc-stop instabilities and improved bead continuity. Its limitations include possible localized heat accumulation at path reversals in complex geometries, while boundary accuracy remains similar to raster strategies.
- Spiral-in: Smooth inward spiraling paths distribute thermal gradients more evenly. Its advantages include improved thermal uniformity for circular or ovoid cross-sections and smoother continuous motion. Its limitation is that it is not readily generalizable to arbitrary polygonal cross-sections.
- Contour: Successive offsets are deposited parallel to the part boundary. Its advantages include high boundary accuracy and regular direction changes that can reduce distortion and anisotropy. Its limitations include the possibility of overfill or underfill in regions where cross-section geometry diverges from the regular contour spacing.

These path strategies should therefore be interpreted not merely as geometric toolpaths, but as thermal-management strategies that determine local reheating, cooling intervals, residual stress distribution, and microstructural uniformity.

Hybrid strategies combining contour perimeter passes with interior zigzag fill, which are widely used in WAAM path planning software, represent the current best practice for geometrically complex parts. The thermal implications of the path strategy are quantified through the thermal diffusivity-based characteristic time:

(13) $$t_{cool}=\frac{L^{2}}{\alpha_{th}}$$

where *L* is the characteristic length of the deposit cross-section, and *α_th_* is the thermal diffusivity. Patterns with shorter characteristic distances between path reversals, such as raster strategies, provide shorter cooling times and lower inter-layer heat accumulation than patterns with longer uninterrupted paths, such as contour paths in large cross-sections.

### 5.2. Inter-Pass Temperature Control and Heat Accumulation

Because deposition path controls local reheating and cooling intervals, path planning must be considered together with inter-pass temperature management. As WAAM builds progress in height, the thermal resistance between the melt pool and the heat sink increases because the deposited material progressively separates the active melt pool from the substrate and because the geometrical heat-sink area does not increase proportionally with deposited volume. In H13 tool-steel walls deposited with pulsed MIG at a controlled inter-layer temperature of 150 °C, Wang et al. [58] attributed the variety of microstructures observed at different locations to the different thermal histories of those regions.

**Figure 9:**
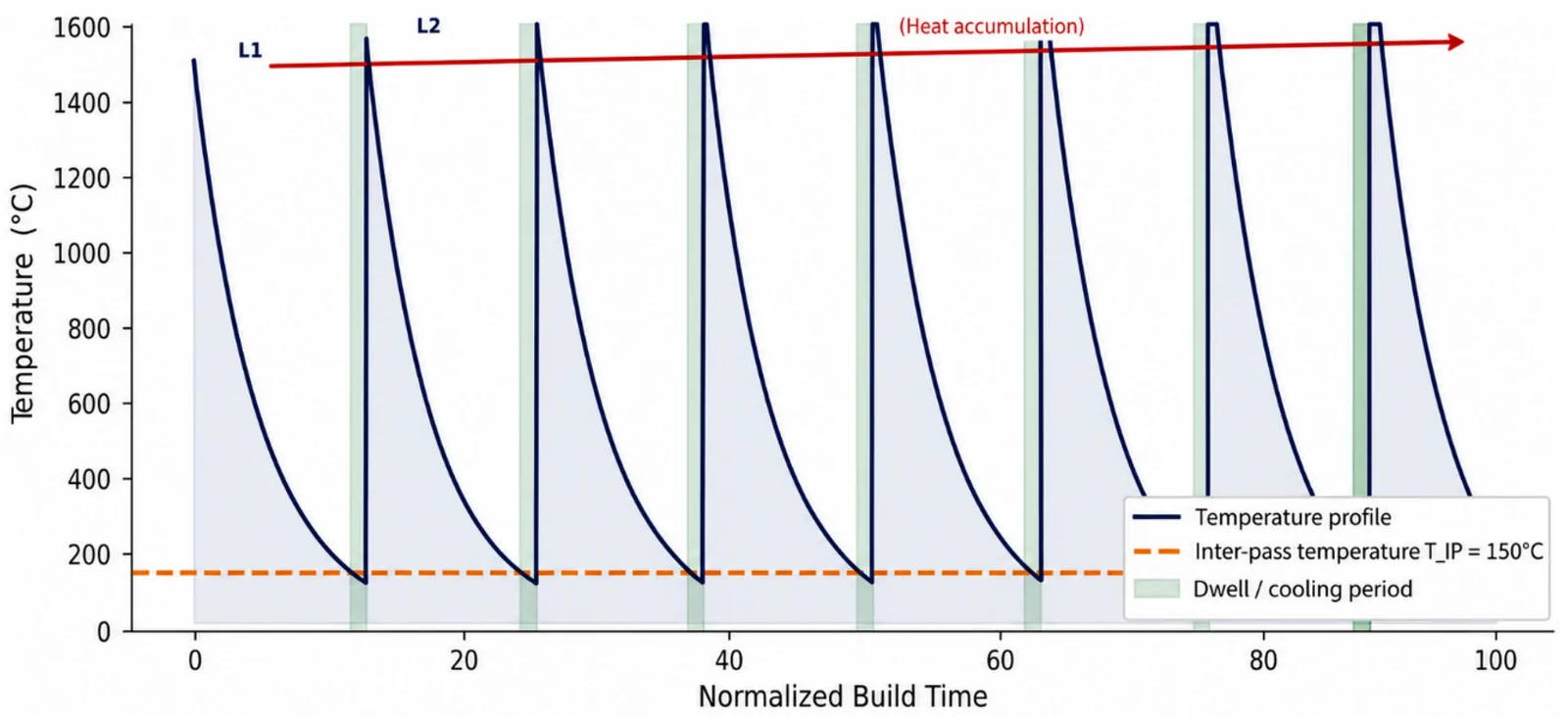
Schematic of repeated thermal cycles at a fixed location in a multi-layer WAAM deposit over normalized build time, showing reheating by successive layers, dwell/cooling periods, the inter-pass temperature threshold T_IP_, and progressive heat accumulation. Conceptual schematic generated with ChatGPT (GPT-5) from author-specified content; no numerical data were reproduced, extracted, or digitized. The trends follow the thermal histories reported for multilayer WAAM in [59,61,62,63].

Two principal inter-pass temperature management strategies exist. The first is fixed dwell time, in which deposition of each new layer is delayed by a fixed time after completion of the previous layer. This approach is simple to implement but does not ensure a consistent thermal state because cooling is non-linear. The second is fixed inter-pass temperature, T_IP_, in which deposition begins only when the deposit surface reaches a target temperature. Montevecchi et al. [59] advocated fixed T_IP_ control as better suited to maintaining thermal consistency. However, Geng et al. [60] cautioned that identifying the appropriate T_IP_ is largely empirical, and the analytical process model of Rios et al. [61] for Ti-6Al-4V deposition showed that the inter-pass temperature must be included to predict layer height and wall width, confirming that the thermal state at the start of each layer is a process variable in its own right. Figure 9 illustrates the thermal history of a multi-layer WAAM deposit. It presents the peak temperature reached in each layer and the gradual heat accumulation with increasing build height. The figure also indicates the inter-pass temperature control trigger, T_IP_, and the dwell cooling periods between successive layers.

**Figure 10:**
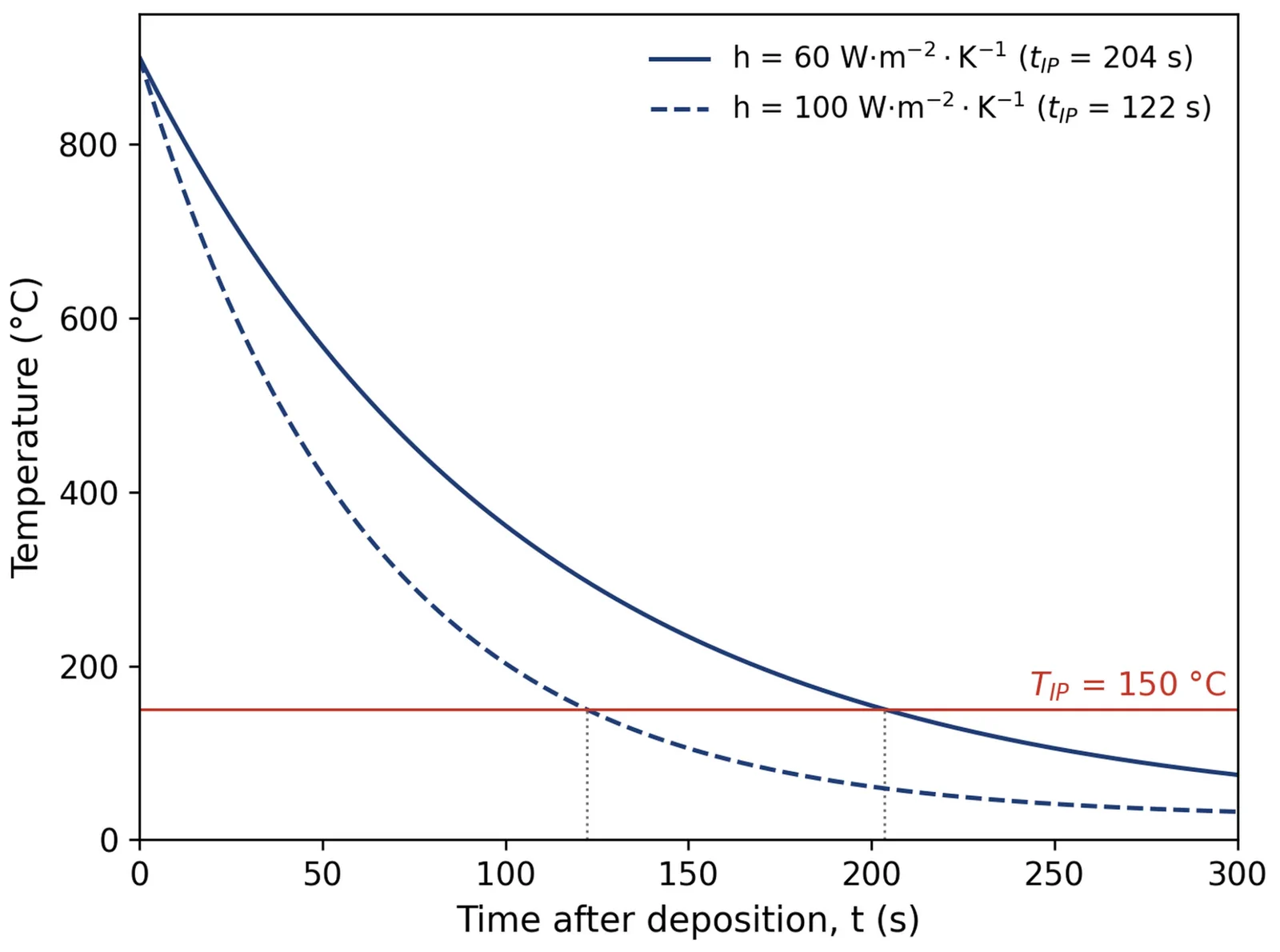
Inter-pass cooling of a steel bead (8 mm × 2 mm × 200 mm; *T_peak_* = 900 °C) calculated with the lumped-capacitance model of Equation (14) for effective heat-transfer coefficients of 60 and 100 W·m^−2^·K^−1^; the calculated times to reach *T_IP_* = 150 °C are 204 and 122 s, respectively. Curves calculated and drawn with Python (Matplotlib 3.10); conduction into previous layers is neglected, so the dwell times are upper-bound estimates.

The inter-pass cooling time t_IP_ to reach a target *T_IP_* from *T_peak_* can be estimated using Newton’s law of cooling for a simplified geometry:

(14) $$t_{IP}=\frac{mc_{p}}{hA_{s}}ln\left(\frac{T_{peak}-T_{\infty }}{T_{IP}-T_{\infty }}\right)$$

where *m* is the mass of the deposit, *c_p_* is the specific heat, *h* is the effective heat-transfer coefficient (convection plus radiation), *A_s_* is the exposed surface area, and *T*_∞_ is the ambient temperature. As an order-of-magnitude example, a steel bead of 8 mm × 2 mm × 200 mm (*m* ≈ 25 g, *c_p_* ≈ 600 J·kg^−1^·K^−1^, and *A_s_* ≈ 2.4 × 10^−3^ m^2^ for the top and side faces) cooling from *T_peak_* ≈ 900 °C to *T_IP_* = 150 °C with h ≈ 60–100 W·m^−2^·K^−1^ gives *t_IP_* ≈ 120–200 s. Because this lumped estimate neglects conduction into the previously deposited layers and the substrate, which is usually the dominant heat-loss path in low walls, it overestimates the dwell time required early in the build; Figure 10 shows the resulting cooling curves. The key point is that *t_IP_* scales with *m*·*c_p_*/(*h*·*A_s_*): as the build grows and the heat-sink path through the wall lengthens, the dwell time needed to reach a fixed *T_IP_* tends to increase, an important scheduling consideration for industrial WAAM process planning.

**Figure 11:**
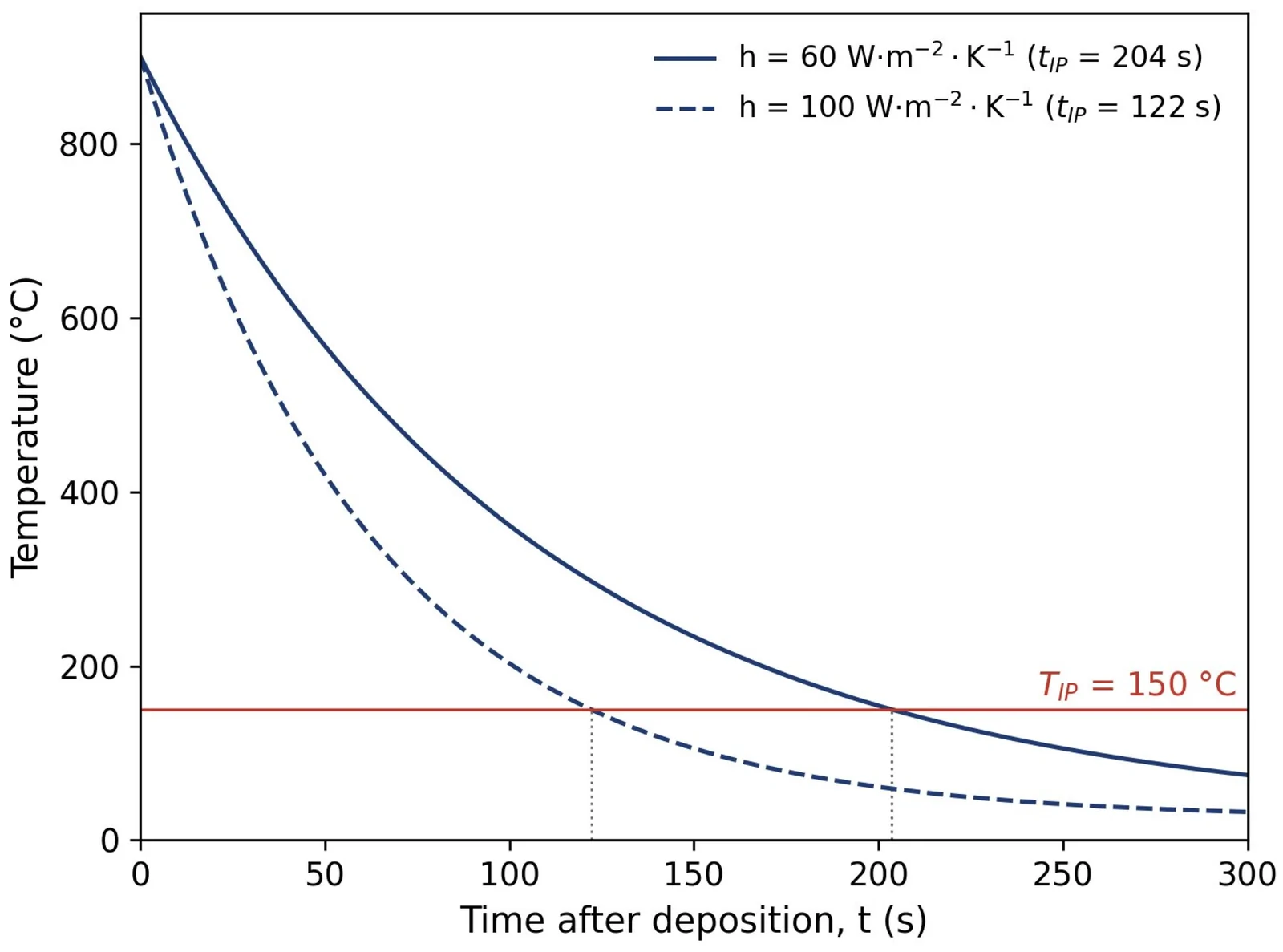
Temperature–time cooling curve used to determine interpass-temperature waiting times in WAAM. Reproduced from Zhai et al. [63] under the Creative Commons Attribution (CC BY 4.0) license.

Figure 9 summarizes schematically how repeated deposition produces rapid reheating, cooling intervals, and progressive heat accumulation relative to an inter-pass temperature threshold, and Figure 11 shows an experimentally derived temperature–time curve of the type used to set inter-pass waiting times [63].

## 6. Welding Metallurgy of WAAM: Melt Pool, Solidification, Phase Transformation and Residual Stress

The process-level variables discussed above ultimately affect WAAM performance through their influence on melt-pool behavior, solidification, phase transformation, and residual stress development.

### 6.1. Melt-Pool Thermodynamics and Heat-Source Modeling

Traditional fusion welding and WAAM are governed by the closely related fundamental thermodynamic and fluid-dynamics principles. In both cases, the arc creates a localized heat source that raises the material above the liquidus temperature T_L_, forming a melt pool that is bounded by the solidification front at T_L_ and the fusion boundary at T_S_ (solidus). The heat flux distribution from the arc is commonly modeled using Goldak’s double-ellipsoidal heat source model [29]:

(15) $$q_{f}\left(x,y,z\right)=\frac{6\sqrt{3}\;f_{f}P}{abc_{f}\pi \sqrt{\pi }}exp\left(-\frac{3x^{2}}{a^{2}}-\frac{3y^{2}}{b^{2}}-\frac{3z^{2}}{c_{f}^{2}}\right)$$

(16) $$q_{r}\left(x,y,z\right)=\frac{6\sqrt{3}\;f_{r}P}{abc_{r}\pi \sqrt{\pi }}exp\left(-\frac{3x^{2}}{a^{2}}-\frac{3y^{2}}{b^{2}}-\frac{3z^{2}}{c_{r}^{2}}\right)$$

where *f_f_* and *f_r_* are energy fractions deposited in the front and rear ellipsoids (*f_f_* + *f_r_* = 2), *q_f_* and *q_r_* are the volumetric heat-flux densities (W·m^−3^) in the front and rear ellipsoids, *P* = ηVI is the effective arc power (W) from Equation (1), *x*, *y*, and *z* are the transverse, depth, and travel-direction coordinates in the frame moving with the arc, and *a*, *b*, *c_f_*, *c_r_* are the semi-axes of the ellipsoidal heat source. Using the linear heat input *H* in place of *P* would make Equations (15) and (16) dimensionally inconsistent. This model captures the asymmetric temperature field around the moving arc with a steeper temperature gradient ahead of the torch and a longer thermal tail behind and has been widely validated in welding thermal analyses [29,64].

Within the melt pool, fluid flow is driven by four competing forces: Marangoni or thermocapillary convection, buoyancy-driven convection, electromagnetic or Lorentz forces, and arc pressure. The Marangoni number Ma characterizes the dominance of surface-tension-driven flow:

(17) $$Ma=\frac{\left|d\gamma /dT\right|\Delta T\;L_{mp}}{\mu \;\alpha_{th}}$$

where *dγ*/*dT* is the temperature coefficient of surface tension (N/m·K), ΔT is the temperature difference across the melt pool surface, *L_mp_* is the melt pool length, and μ is the dynamic viscosity. Under WAAM conditions the melt-pool flow is driven mainly by the surface-tension gradient on the pool surface, and heat transport by convection greatly exceeds that by conduction. Using three-dimensional heat-transfer and fluid-flow modeling of H13 tool-steel deposits, Ou et al. [65] calculated Péclet numbers of approximately 100 and found that neglecting convection made the calculated pool width 13% smaller than that measured, confirming that convection is the main heat-transfer mechanism inside the melt pool.

The Reynolds number *Re* = *ρ_l_* · *v_fl_* · *L_mp_*/*μ* of the melt-pool flow characterizes the ratio of inertial to viscous forces; together with Ma, it governs how strongly the flow redistributes solute and affects gas escape and porosity formation.

### 6.2. Solidification and the G/R Framework

The microstructural outcome of solidification is controlled by two parameters: the temperature gradient *G* (K/m) at the solidification front and the solidification velocity *R* (m/s). Their ratio *G*/*R* governs the stability of the solid–liquid interface and hence the resulting solidification mode, as established by the constitutional undercooling criterion [15]:

(18) $$\frac{G}{R}\geq \frac{-m_{L}C_{0}\left(1-k\right)}{kD_{L}}=\frac{\Delta T_{0}}{D_{L}}$$

where *m_L_* is the liquidus slope (K/wt%; negative for k < 1), *C*_0_ is the nominal alloy composition (wt%), k is the equilibrium partition coefficient, *D_L_* is the solute diffusivity in the liquid (m^2^/s), and *ΔT*_0_ is the equilibrium solidification range of the alloy (*K*). When *G*/*R* falls below this threshold, the planar interface becomes unstable and transitions sequentially to cellular, columnar-dendritic, and equiaxed-dendritic morphologies as *G*/*R* decreases further [15,16]. The transition from columnar to equiaxed growth (the CET) occurs approximately when:

(19) $$\frac{G}{R^{1/2}}<C_{CET}$$

where *C_CET_* is a material-dependent constant [16]. In WAAM, directional heat extraction through the substrate and previously deposited material generally promotes columnar microstructures unless active intervention, such as grain refiners, arc oscillation, inter-layer deformation, or external field assistance, is applied.

The product *G*·*R* equals the local cooling rate *Ṫ* (K/s) at the solidification front and governs the primary dendrite arm spacing (PDAS) *λ*_1_:

(20) $$\lambda_{1}=a{\left(G\cdot R\right)}^{-n}=a\;{\dot{T}}^{-n}$$

where *a* and *n* are alloy-dependent constants (*n* ≈ 0.25–0.50) [15,16]. This relationship is important for WAAM because finer PDAS, achieved by higher *G*·*R* cooling-rate products, can reduce inter-dendritic segregation and improve mechanical properties. In WAAM, however, *G*·*R* may decrease as build height increases because the thermal path to the substrate becomes longer and heat accumulation increases, causing PDAS to increase from base to top of the deposit, a source of microstructural heterogeneity; finite element analysis of GMAW-based circular thin walls showed that the temperature gradient in the melt pool decreases as the deposition height increases [66].

The significance of the *G*/*R* framework in WAAM is therefore not only predictive but diagnostic, because it explains why identical nominal arc parameters can produce different microstructures at different build heights as the thermal boundary condition evolves.

### 6.3. Welding Versus WAAM: Repeated Thermal Cycling and Microstructural Consequences

While WAAM and welding share the physics described above, they differ in several important ways that have direct consequences for microstructure and properties:

- Repeated thermal cycling: In single-pass welding, each material point generally experiences one dominant heating-cooling cycle. In WAAM, each point experiences multiple thermal cycles from the deposition of subsequent layers, with the number of cycles depending on build height and layer thickness. Each cycle reheats the material to a peak temperature T_peak_ that decreases with distance from the torch. These repeated cycles drive: (a) grain growth, following the isothermal kinetic relation $\bar{d}$*^n^* − $\bar{d}$_0_*^n^* = *K*_0_·*t*·*exp*[−*Q_g_*/(*R_g_*·*T*)] (Table 4), with n = 2 for ideal parabolic growth and n > 2 when boundaries are pinned, integrated over the thermal history, (b) precipitation/dissolution of secondary phases, (c) recovery and partial recrystallization, and (d) microstructural banding due to alternating coarse/fine zones corresponding to T_peak_ above and below the recrystallization temperature *T_rex_*.
- Thermal gradient directionality: In welding, the dominant heat extraction direction is often lateral into the parent plates, whereas in WAAM, heat is extracted primarily downward through the substrate and previously deposited material, especially in the early build stages. This promotes epitaxial columnar grain growth along the build direction (BD). The result is often a strong crystallographic texture, typically <001> parallel to BD in cubic metals, which may persist across the build height unless disrupted by allotropic transformations, as in Ti-6Al-4V, where the β→α transformation partially randomizes texture, or by active grain refinement strategies [67].
- Chemical composition freedom: In single-pass welding of two bulk components with filler wire, the resulting deposit chemistry is strongly influenced by dilution with the base material. In WAAM, material is deposited onto a flat substrate or prior layer, allowing the deposit chemistry to be varied spatially by switching wire feedstock or using multiple feed wires, thereby enabling functionally graded structures with tailored property distributions; twin-wire WAAM has been used to fabricate intermetallic alloys and functionally graded materials [35].
- Process automation and precision: Welding may be manual, mechanized, or automated, whereas WAAM is typically computer-controlled, enabling improved reproducibility of melt-pool placement, layer thickness, and deposition trajectory compared with manual welding and comparable to advanced automated welding systems [16].
- Melt pool aspect ratio and penetration: Welding often requires a relatively high depth-to-width melt-pool ratio to achieve joint penetration, whereas WAAM usually requires controlled remelting of the prior layer rather than full plate penetration. This generally produces shallower melt pools and different *G*/*R* distributions at the solidification front, which can alter dendritic growth, fusion-boundary morphology, and inter-layer bonding compared with conventional welding at equivalent power levels.

Several of the characteristics listed above are shared with multi-pass welding. Thick-section joints, such as reactor pressure vessel welds that may require hundreds of passes, also experience repeated reheating, heat accumulation, tempering or re-transformation of earlier passes, and residual stress build-up, and the metallurgy of reheated multi-pass weld metal applies directly to WAAM [16,19]. The distinctive features of WAAM arise from its geometry and boundary conditions rather than from repetition itself. (i) In a joint, each pass is bounded by the groove faces of the parent plates, which act as lateral heat sinks and epitaxial substrates, whereas a WAAM wall is free-standing, so heat is conducted downward through a lengthening path of previously deposited material and lost from two free side surfaces. (ii) In a joint, the bead sequence, bead placement, and groove geometry change the direction of the thermal gradient from pass to pass, whereas in a WAAM wall the fusion boundary advances upward in the same direction layer after layer, so the thermal gradient remains aligned with the build direction while its magnitude decreases with height [66]. (iii) The deposit forms the whole component rather than a joint between wrought members, so the reheated as-deposited microstructure and its build-height gradient determine the properties of the entire part. (iv) Inter-pass conditions in WAAM are usually set by productivity and geometric stability rather than by a qualified welding procedure, and they change as the build grows.

The different microstructures arising from these distinct thermal conditions can be described conceptually across the build height. Moving from the substrate toward the top of a WAAM build, one may observe a gradient from a remelted or refined near-substrate region, through a thermally cycled mid-build region characterized by banded microstructures, to a columnar-dominated near-top region that has experienced the fewest reheating cycles. Wang et al. [58] likewise reported location-dependent microstructures in H13 walls, although the hardness remained within 300–360 HV from top to bottom and the as-deposited tensile properties were anisotropic.

### 6.4. Phase Transformations and Residual Stress Development

In steels and titanium alloys, solid-state phase transformations during cooling from the melt pool strongly influence the final room-temperature microstructure. In single-pass welding of ferritic steels, the weld metal cools from the austenite (γ) field through the Ac_3_ temperature and transforms to ferrite morphologies such as allotriomorphic ferrite, Widmanstätten ferrite, acicular ferrite, bainite, or martensite depending on cooling rate and alloy composition, as represented by continuous cooling transformation (CCT) diagrams [16]. In WAAM, the same transformation may occur during the first cooling cycle; however, subsequent layers reheat the prior deposit to peak temperatures that may partially or fully austenitize it again, driving repeated transformation cycling that progressively homogenizes the microstructure in mid-build regions while leaving the topmost layers in a more as-deposited transformation state [12,40].

For Ti-6Al-4V, the dominant high-temperature phase is body-centered cubic β, which transforms on cooling to hexagonal close-packed α, forming lamellar colonies or Widmanstätten α, or to α′ martensite at higher cooling rates. In WAAM, the primary β columnar grains form by epitaxial solidification and persist through the β-field on cooling. The β → α transformation then partitions the columnar β into a microstructure of α laths, whose morphology, including coarse lamellae, fine basketweave, or acicular martensite, is controlled by the local cooling rate through the β transus [13,62,68]. Repeated reheating below the β transus in subsequent layers partially homogenizes this microstructure but does not fully eliminate prior-β columnar grain inheritance, a limitation that hybrid deformation strategies such as rolling and FSP are designed to overcome [69].

In aluminum alloys, which lack allotropic transformations, the solid-state microstructural evolution in WAAM is controlled primarily by precipitation, dissolution, coarsening kinetics, and grain-boundary migration. Repeated thermal cycling can dissolve strengthening precipitates, such as θ′-Al_2_Cu in 2xxx series alloys and β″-Mg_2_Si in 6xxx series alloys, in the heat-affected region of each deposited layer, creating over-aged or solution-annealed zones that degrade local strength. This precipitate heterogeneity, characterized by alternating zones of fresh precipitation and dissolution, is one of the primary sources of the banded hardness profiles characteristic of aluminum WAAM builds [70]. In addition to phase transformation, residual stress development is another major metallurgical consequence of repeated WAAM thermal cycling.

**Figure 12:**
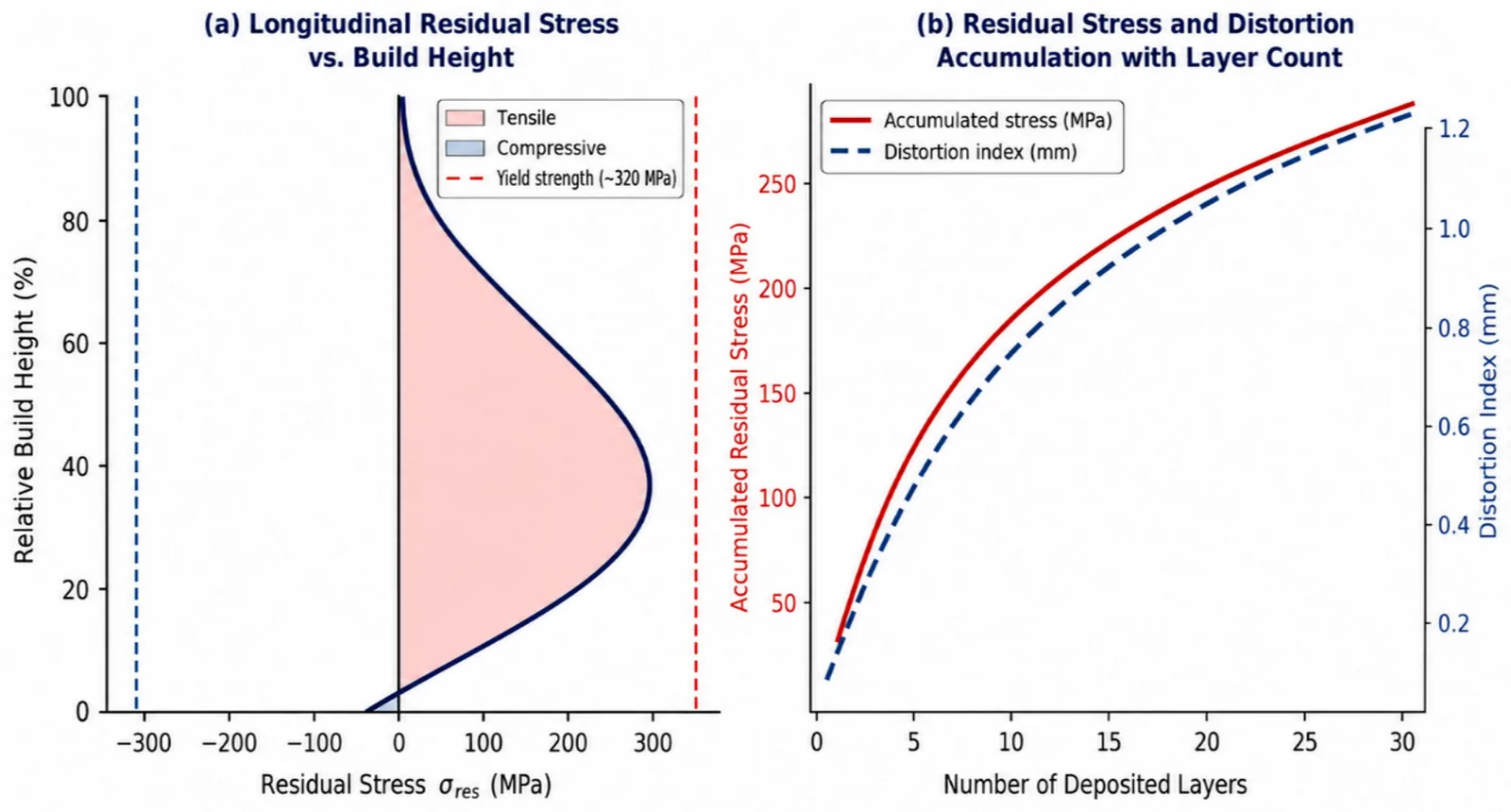
Conceptual schematic generated with ChatGPT (GPT-5) from author-specified content: (**a**) longitudinal residual-stress profile through the WAAM build height relative to the material yield strength; (**b**) accumulation of residual stress and distortion with increasing number of deposited layers. No graphical content or numerical data were reproduced or digitized; the concept is supported by the cited residual-stress studies [25,26].

Residual stresses in WAAM arise from the differential thermal contraction of the cooling deposit under structural constraint. The thermal strain ε_th_ in a deposited bead cooling from *T_peak_* to ambient *T*_0_ is:

(21) $$\varepsilon_{th}=\alpha_{CTE}\left(T_{peak}-T_{0}\right)$$

where *α_CTE_* is the coefficient of thermal expansion (K^−1^). If this strain is partially or fully constrained by the surrounding cooler material, a corresponding elastic or elastic-plastic stress develops. The fully constrained elastic thermal stress, which serves as an upper-bound indicator of the residual stress in single-pass welding, is [16]:

(22) $$\sigma_{res}\approx \frac{E\;\alpha_{CTE}\;\Delta T}{1-\nu }$$

where *E* is Young’s modulus (Pa) and ν is Poisson’s ratio. For a steel deposit with *E* ≈ 210 GPa, *α_CTE_* ≈ 12 × 10^−6^ K^−1^, *ν* ≈ 0.3, and *ΔT* ≈ 500–800 K, Equation (22) gives approximately 1.8–2.9 GPa, several times the yield strength of typical WAAM steel deposits. This elastic stress cannot be sustained: the constrained material yields during cooling, and the residual stress saturates at a level governed by the temperature-dependent yield strength, which is why measured longitudinal residual stresses in as-deposited WAAM walls reach the flow strength of the alloy; in 2319 aluminum walls, neutron diffraction showed such stresses along the full build height [25]. Equation (22) is therefore useful to indicate whether plastic flow will occur, i.e., whether *E*·*α_CTE_*·*ΔT*/[(1 − *ν*)·*σ_y_*] > 1, not to predict the magnitude of the residual stress. Figure 12 shows the residual stress and distortion behavior in WAAM. Figure 12a shows the longitudinal residual stress profile along the build height and compares it with the material yield strength. Figure 12b shows that residual stress and distortion increase gradually as more layers are deposited. In WAAM, however, the cumulative effect of multiple deposited layers progressively increases the stored elastic strain energy, meaning that the residual stress state in a tall build cannot be predicted reliably from single-pass welding models alone and requires three-dimensional thermo-mechanical simulation.

Vimal et al. [70] identified the hierarchy of WAAM residual stress consequences: when *σ_res_* > *σ_UTS_*, solidification or delamination cracking may occur; when *σ_y_* < *σ_res_* < *σ_UTS_*, plastic deformation and distortion may develop; when *σ_res_* < *σ_y_*, residual stress may still reduce fatigue life by increasing the mean stress in cyclic loading.

## 7. Microstructural Evolution and Defect Formation in WAAM

The metallurgical mechanisms discussed in Section 6 provide the basis for understanding the microstructural gradients and defects that develop during WAAM.

### 7.1. Columnar Grain Growth, Epitaxy and Build-Height Heterogeneity

The macrostructure of WAAM deposits is commonly characterized by epitaxial columnar grains that nucleate on the solid substrate or prior layer and grow preferentially along the maximum thermal gradient, often aligned with the build direction (BD). This epitaxial growth occurs because the nucleation barrier ΔG* for heterogeneous nucleation on an existing grain is zero when the growth direction is crystallographically aligned with the thermal gradient [15]:

(23) $$\Delta G_{epitaxial}^{*}=0$$

compared to ΔG*_homogeneous_ = 16*πγ_SL_*^3^/(3*ΔG_V_*^2^) for homogeneous nucleation. This energy advantage of epitaxial growth explains why columnar grains spanning multiple deposited layers are frequently observed in WAAM of cubic metals such as steels, aluminum alloys, and nickel alloys. Only conditions that provide sufficient constitutional undercooling ahead of the solidification front or that introduce potent nucleation sites can promote the columnar-to-equiaxed transition (CET) [16].

**Figure 13:**
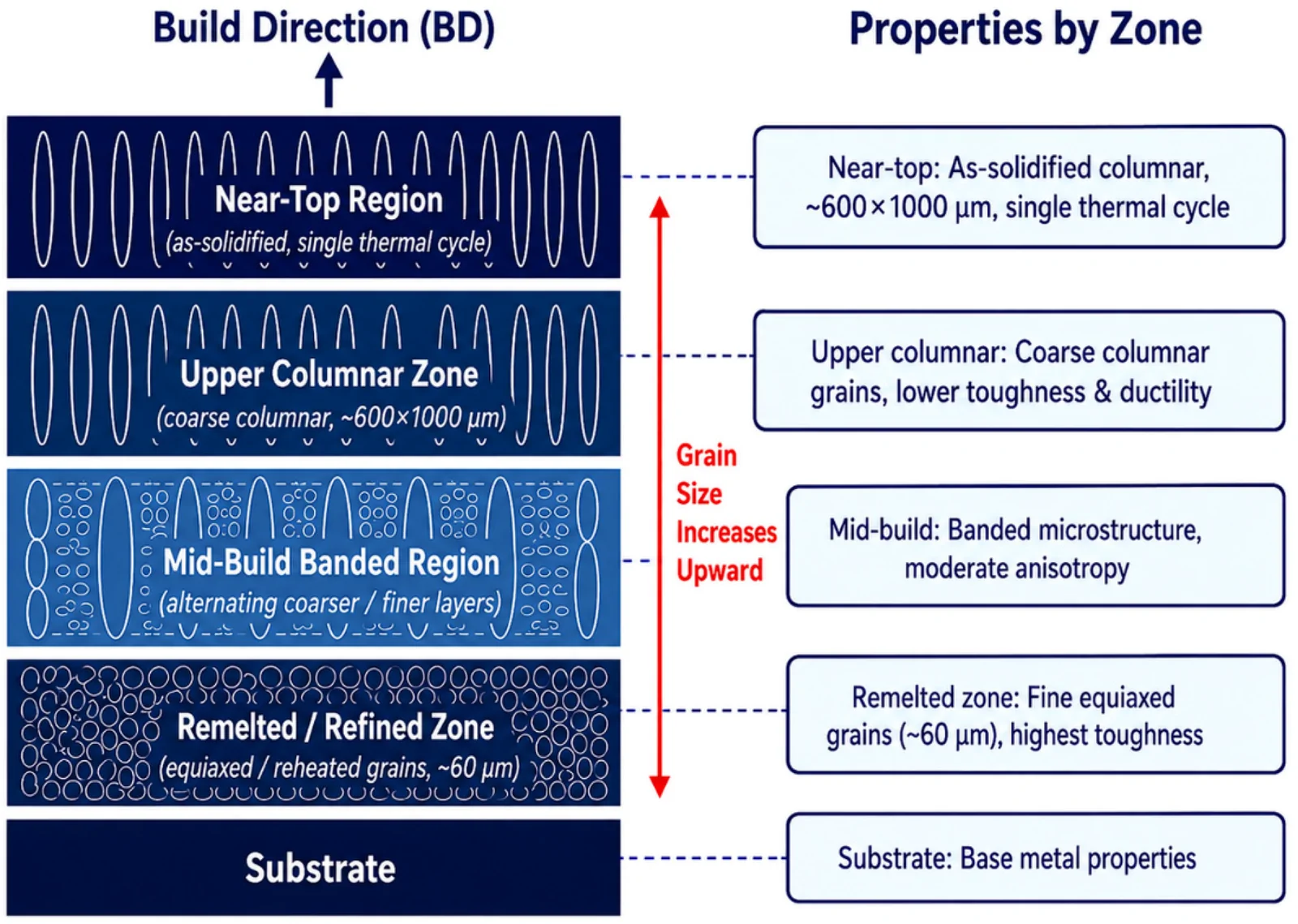
Schematic of the microstructural gradient from substrate to build top in a WAAM deposit. Zone-dependent grain morphology evolves from fine equiaxed grains in remelted near-substrate regions through a mixed columnar–equiaxed transition zone to coarse columnar grains in unreheated near-top layers, governed by the local *G*/*R* ratio at each solidification front. Conceptual schematic generated with ChatGPT (GPT-5) from author-specified content; no numerical data or graphical content were reproduced, adapted, or digitized from a published source. The concept is supported by prior studies on WAAM build-height heterogeneity, epitaxial columnar grain growth, and remelting-driven microstructural variation [2,23,33,34].

The resulting microstructure varies as a function of build position. In the remelted zone immediately adjacent to the fusion boundary with the substrate, grains nucleate epitaxially on the substrate grain structure and grow rapidly into the melt pool. In the mid-build region, columnar grains may span multiple layers, periodically interrupted by the remelted zone of each subsequent pass but generally maintaining directional continuity. In the near-top region, typically the topmost few layers, depending on layer thickness and thermal history, grains are less affected by reheating from above and therefore may exhibit a coarser, more as-solidified morphology [12,40]. Figure 13 illustrates the microstructural gradient from the substrate to the build top in a WAAM deposit. The near-substrate region contains fine equiaxed grains due to repeated remelting and reheating. In contrast, the near-top region develops coarse columnar grains because these layers experience less reheating after deposition.

**Figure 14:**
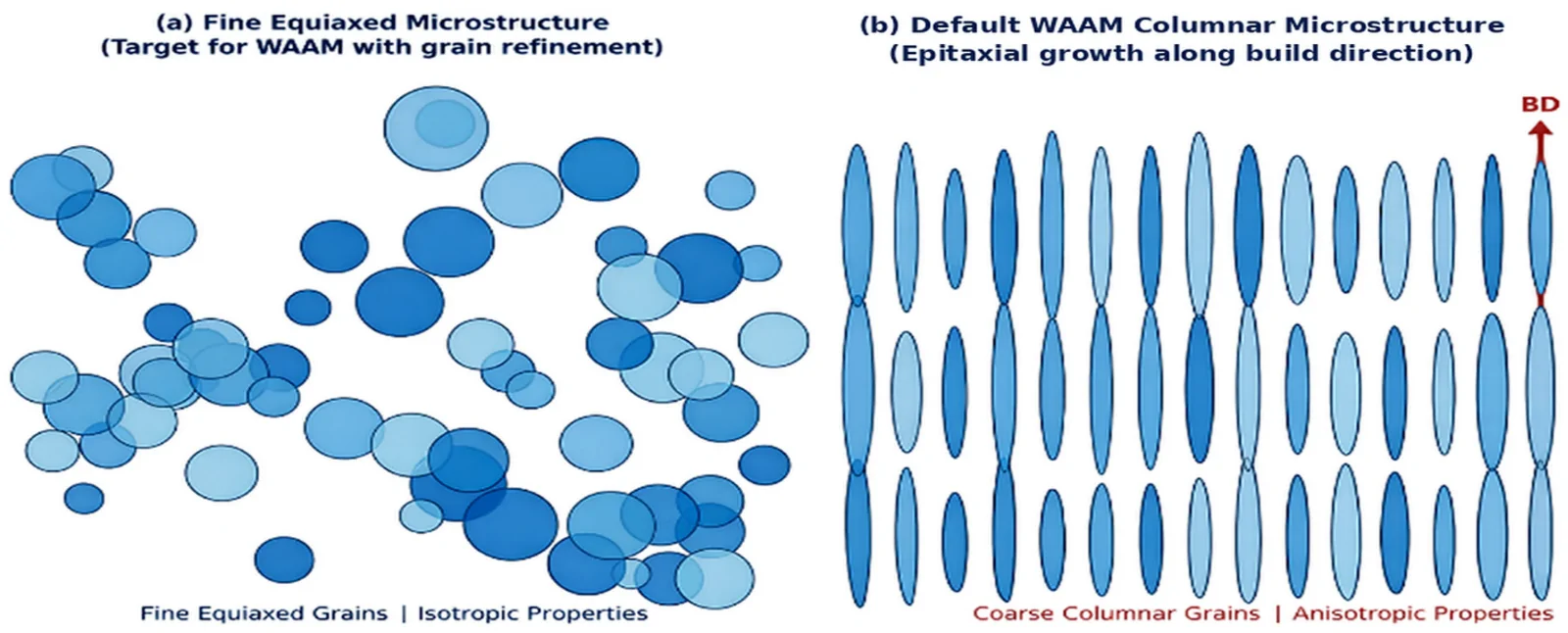
Schematic comparison of (**a**) the fine equiaxed microstructure targeted through grain refinement strategies and (**b**) the default columnar microstructure produced by epitaxial solidification in WAAM along the build direction. Conceptual schematic generated with ChatGPT (GPT-5) from author-specified content; no numerical data or published graphical content were reproduced.

Dickens et al. [12,40] documented this progression in mild steel WAAM: mid-wall regions showed large equiaxed ferrite (α) and pearlite (α + Fe_3_C) with grain sizes of approximately 60 µm, consistent with multiple reheating cycles driving partial recrystallization, while the topmost layers exhibited much coarser columnar grains of approximately 600 µm × 1000 µm. This top-to-bottom gradient in grain size is not merely morphological; it can produce measurable gradients in Vickers hardness of 20–40 HV and tensile strength variations of 10–25% across the build height, which must be considered in structural design [12,32]. Figure 14 compares the desired refined microstructure with the typical WAAM solidification structure. Figure 14a depicts the fine equiaxed grains targeted through grain refinement strategies. Figure 14b illustrates the default columnar grain structure formed by epitaxial solidification along the build direction.

**Figure 15:**
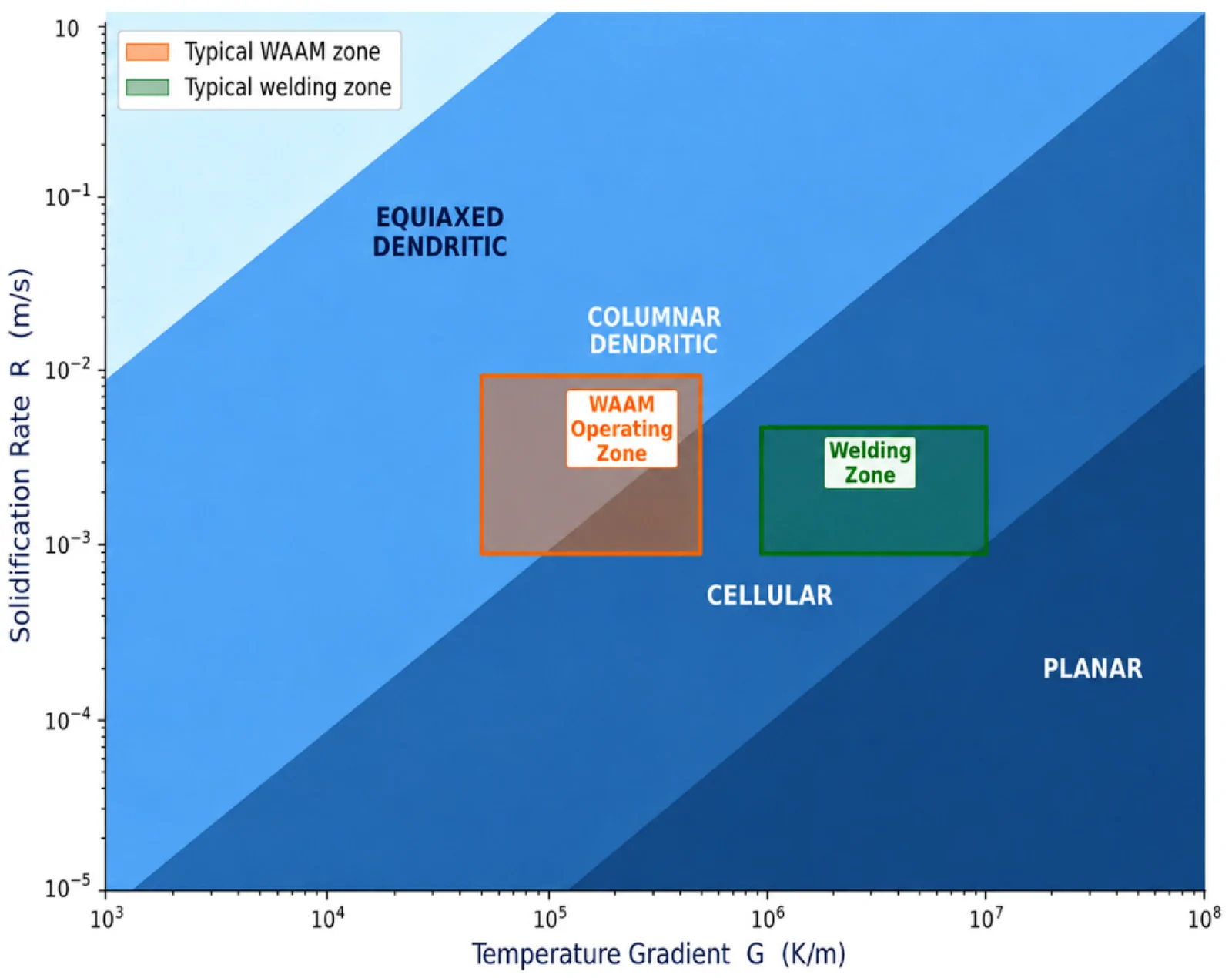
Solidification map showing the relationship between thermal gradient G, solidification rate R, and expected solidification morphology, including planar, cellular, columnar-dendritic, and equiaxed-dendritic regimes. Typical WAAM and single-pass welding operating zones are shown schematically. The figure is a conceptual schematic, generated with ChatGPT (GPT-5) from author-specified content, based on classical solidification-map concepts and AM solidification analyses [15,33,71,72]; no numerical data were extracted, and no published solidification map was reproduced or adapted.

Beyond the qualitative description of epitaxial growth, the *G*/*R* framework provides a quantitative basis for predicting these grain morphologies. The primary scientific tool for predicting WAAM solidification microstructure is the solidification map, a plot of *G* versus *R*, or *G*/*R* versus *G*·*R*, that delineates the boundaries between planar, cellular, columnar-dendritic, and equiaxed-dendritic regimes. Figure 15 illustrates the solidification map based on the temperature gradient (*G*) and solidification growth rate (*R*). The map identifies the planar, cellular, columnar-dendritic, and equiaxed-dendritic regimes. It also indicates the typical operating zones for WAAM and single-pass welding.

For a given alloy system, these boundaries are calculated from Equation (18) and the dendrite stability criteria [15,16]. In WAAM, the *G* and *R* operating points vary continuously around the melt pool perimeter: *G* is highest and *R* is lowest at the fusion boundary (where solidification is just established), yielding high *G*/*R* values that favor planar or cellular growth; toward the pool center, *G* decreases and *R* increases, producing *G*/*R* values more characteristic of columnar-dendritic growth. This spatial variation produces the mixed microstructures, such as cellular regions near the fusion boundary and columnar-dendritic regions in the melt-pool interior, which are characteristic of many WAAM deposits [16,65].

A practical implication is that increasing the travel speed v at a fixed arc power *P* changes *G* and *R* simultaneously. At the trailing boundary of the melt pool, *R* = *v*·*cos φ*, where *φ* is the angle between the travel direction and the normal to the local solidification front, so *R* varies from approximately *v* at the pool centerline to nearly zero at the fusion boundary, whereas G depends on the melt-pool shape and the thermal boundary conditions [16]. Using three-dimensional heat-transfer and fluid-flow modeling of WAAM deposits, Ou et al. [65] showed that a faster travel speed increases the cooling rate *G*·*R* and decreases *G*/*R*. These two changes act on different aspects of the solidification structure and must be interpreted separately. The increase in *G*·*R* reduces the primary dendrite arm spacing according to Equation (20), i.e., it refines the dendritic length scale. The decrease in *G*/*R* reduces the stability of the solidification front and moves the operating point toward columnar-dendritic growth and, when sufficient constitutional undercooling and nucleation sites are available, toward the columnar-to-equiaxed transition (Equations (18) and (19)); this is a change in solidification morphology, not an increase in dendrite size. A faster travel speed should therefore not be equated with dendritic coarsening, and its net effect on a given deposit must be evaluated with thermal simulation because the melt-pool geometry changes at the same time.

### 7.2. Porosity, Solidification Cracking and Delamination

Porosity is one of the most prevalent and mechanistically complex defects in WAAM, particularly in aluminum alloys. The dominant mechanism, hydrogen porosity, arises from the dramatic change in hydrogen solubility at the melting point [42,73]. For aluminum, hydrogen solubility drops from approximately 0.65 mL/100 g in the liquid state to approximately 0.034 mL/100 g in the solid state at the melting point (a ratio of approximately 19:1) [16]. This discontinuity drives hydrogen rejection from the advancing solidification front: if the local hydrogen concentration *C_H_* exceeds the solubility in the solid *C_H_*^sol^ and the solidification rate is sufficient to entrap rejected hydrogen, gas pores can nucleate and grow.

Pore nucleation is governed by Sieverts’s law [16]:

(24) $$\left[H\right]=K_{S}\;p_{H_{2}}^{1/2}$$

where [*H*] is the hydrogen content in wt ppm, *K_S_* is the Sieverts’s constant (temperature-dependent), and $p_{H_{2}}$ is the partial pressure of hydrogen in the atmosphere. In WAAM, the partial pressure of hydrogen in the shielding environment may arise from moisture contamination on wire and substrate surfaces and from gas-line contamination, which can be sufficient to produce hydrogen contents above the solid solubility threshold.

The pore escape criterion is also important: a hydrogen pore of radius *r_p_* in the melt pool will escape to the surface before solidification only if the Stokes velocity *v_Stokes_* exceeds the solidification front velocity *R* [16]:

(25) $$v_{Stokes}=\frac{2r_{p}^{2}\left(\rho_{l}-\rho_{g}\right)g}{9\mu_{l}}$$

For hydrogen pores in liquid aluminum (*ρ_l_* ≈ 2.4 × 10^3^ kg·m^−3^, *μ_l_* ≈ 1 × 10^−3^ Pa·s), Equation (25) gives *v_Stokes_* ≈ 0.13 mm/s for *r_p_* = 5 µm, ≈ 0.5 mm/s for *r_p_* = 10 µm, and ≈ 13 mm/s for *r_p_* = 50 µm. Because typical solidification rates in WAAM are *R* ≈ 1–10 mm/s, pores smaller than approximately 10–20 µm rise more slowly than the solidification front advances and are likely to be entrapped, whereas pores of several tens of micrometers can escape if they nucleate early enough. This analysis explains why bead geometry matters: a narrow, deep bead requires pores to travel longer distances to escape, increasing entrapment probability. Optimizing bead geometry toward wider and shallower profiles through appropriate current, travel speed, and transfer-mode control can reduce hydrogen porosity [16]. Other process variables also matter: in WAAM of AlSi5 (AW4043), a higher shielding-gas flow rate increased porosity because forced convection accelerated melt-pool solidification and limited pore escape [42], whereas for 21 AA2319 panels, porosity was not strongly dependent on CMT mode, wire feed speed, or the ratio of wire feed speed to travel speed within the tested ranges but varied significantly between wire batches, reflecting differences in wire surface finish [43].

**Figure 16:**
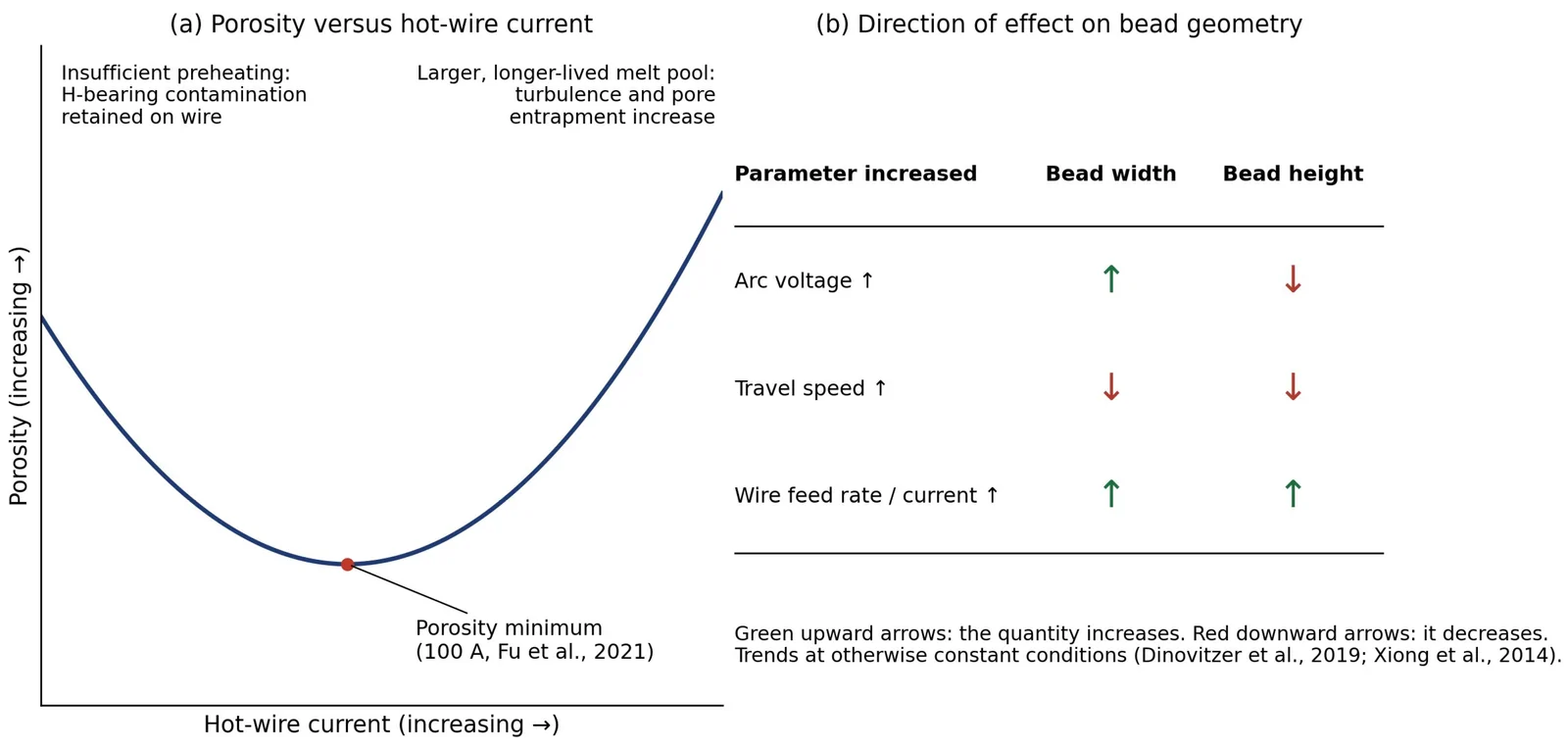
Qualitative trends in aluminum WAAM and GMAW bead geometry: (**a**) porosity versus hot-wire current, with the minimum reported at 100 A by Fu et al. [44]; (**b**) direction of the effect of arc voltage, travel speed, and wire feed rate on bead width and height [38,39]. Schematic drawn with Python (Matplotlib 3.10); no numerical data are plotted.

Fu et al. [44] reported a non-monotonic relationship between hot-wire current and porosity in hot-wire arc additive manufacturing of aluminum, with a minimum at 100 A. A plausible interpretation is that at lower hot-wire currents insufficient preheating leaves hydrogen-bearing surface contamination on the wire; whereas above 100 A, the enlarged melt pool increases Marangoni turbulence and extends the solidification time, both of which can increase pore entrapment. The minimum porosity at approximately 100 A corresponds to the optimal balance between pre-heating, which reduces hydrogen sources, and melt-pool size, which governs pore escape probability. Figure 16 summarizes these trends qualitatively: panel (a) shows the non-monotonic dependence of porosity on hot-wire current, and panel (b) shows the direction in which arc voltage, travel speed, and wire feed rate change bead width and height.

Solidification cracking (hot cracking) occurs when tensile thermal stresses develop across a partially solidified film of eutectic liquid between growing dendrites, rupturing the film before it achieves full solidification. The susceptibility to solidification cracking is quantified by the brittleness temperature range (BTR):

(26) $$BTR=T_{L}-T_{S}^{*}$$

where Ts* is the temperature at which the solid fraction (typically *f_s_* > 0.9) is sufficient to bridge the interdendritic liquid and resist tearing. Alloys with a wide BTR, including heat-treatable aluminum alloys (2xxx and 7xxx series), are more prone to solidification cracking [16]. Coarse columnar grains aggravate this tendency because they concentrate solute segregation and strain on fewer, longer interdendritic liquid films, and alloys with a high *α_CTE_* experience a higher thermal strain rate, *ε̇_th_* = *α_CTE_*·(*dT*/*dt*), across the mushy zone for a given cooling rate [16].

**Figure 17:**
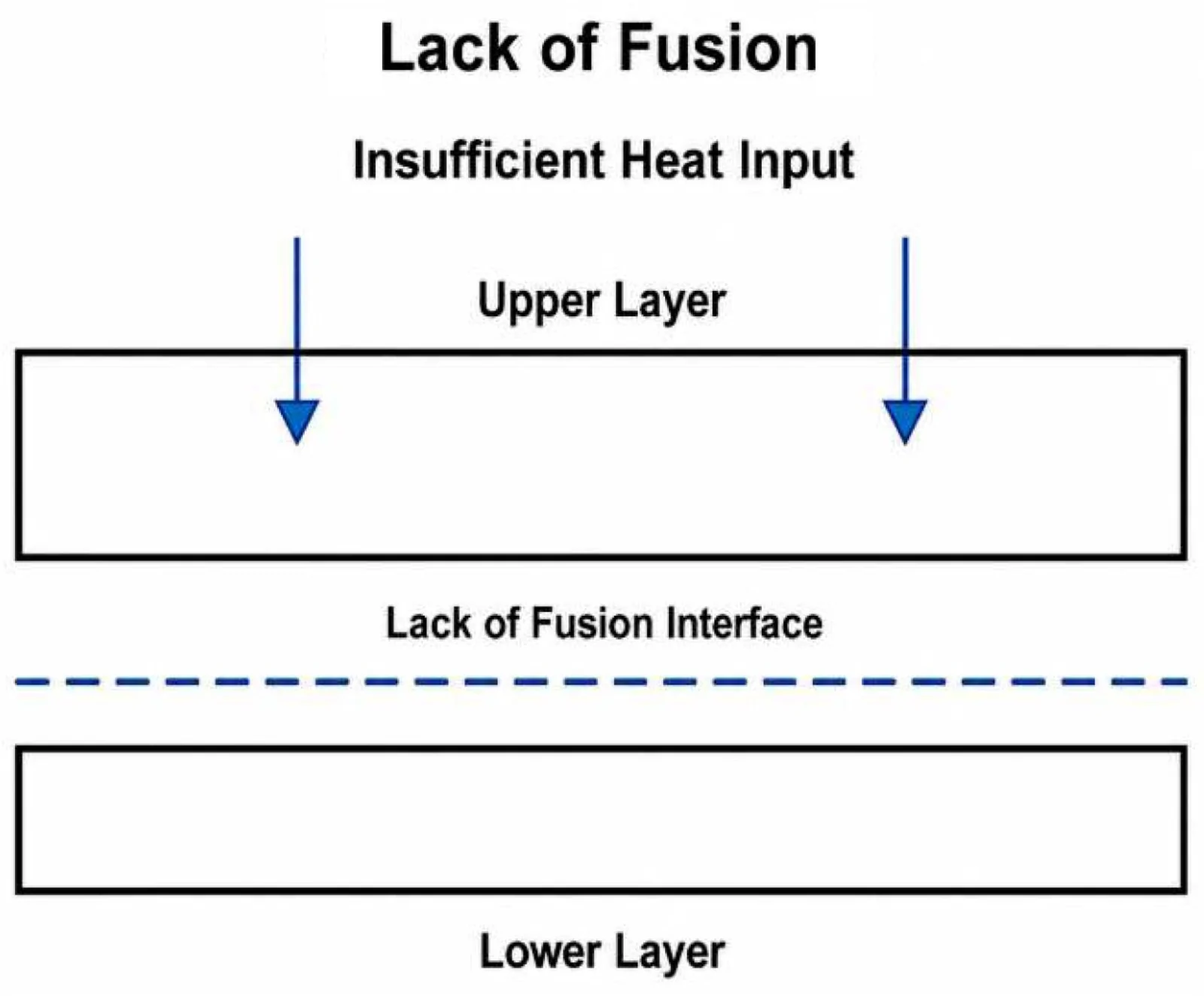
Conceptual schematic, generated with ChatGPT (GPT-5) from author-specified content, of lack-of-fusion defect formation in WAAM: insufficient heat input prevents adequate remelting of the prior layer surface, leaving an unbonded interface (dashed line) between the upper and lower layers. No graphical content was reproduced; the mechanism is supported by the cited study [70].

Delamination, the separation of adjacent deposited layers, occurs when the metallurgical bond formed by inter-layer remelting is insufficient to sustain the residual stresses that develop during subsequent cooling. The condition for delamination is:

(27) $$\sigma_{res}>\sigma_{bond}$$

where *σ_bond_* is the bond strength of the partially remelted interface. Under-remelting, insufficient heat input to the prior layer surface, excessive inter-pass oxidation, or unfavorable inter-pass thermal conditions can reduce *σ_bond_* and increase delamination susceptibility [70]. Figure 17 illustrates the formation of lack-of-fusion defects in WAAM. Insufficient heat input prevents proper remelting of the previous layer surface, leaving an unbonded interface between the upper and lower layers. This unbonded region, marked by the dashed line, becomes the main source of delamination when sufficient bond strength, *σ_bond_*, is not established.

### 7.3. Summary of Defect Mechanisms and Governing Relationships

**Figure 18:**
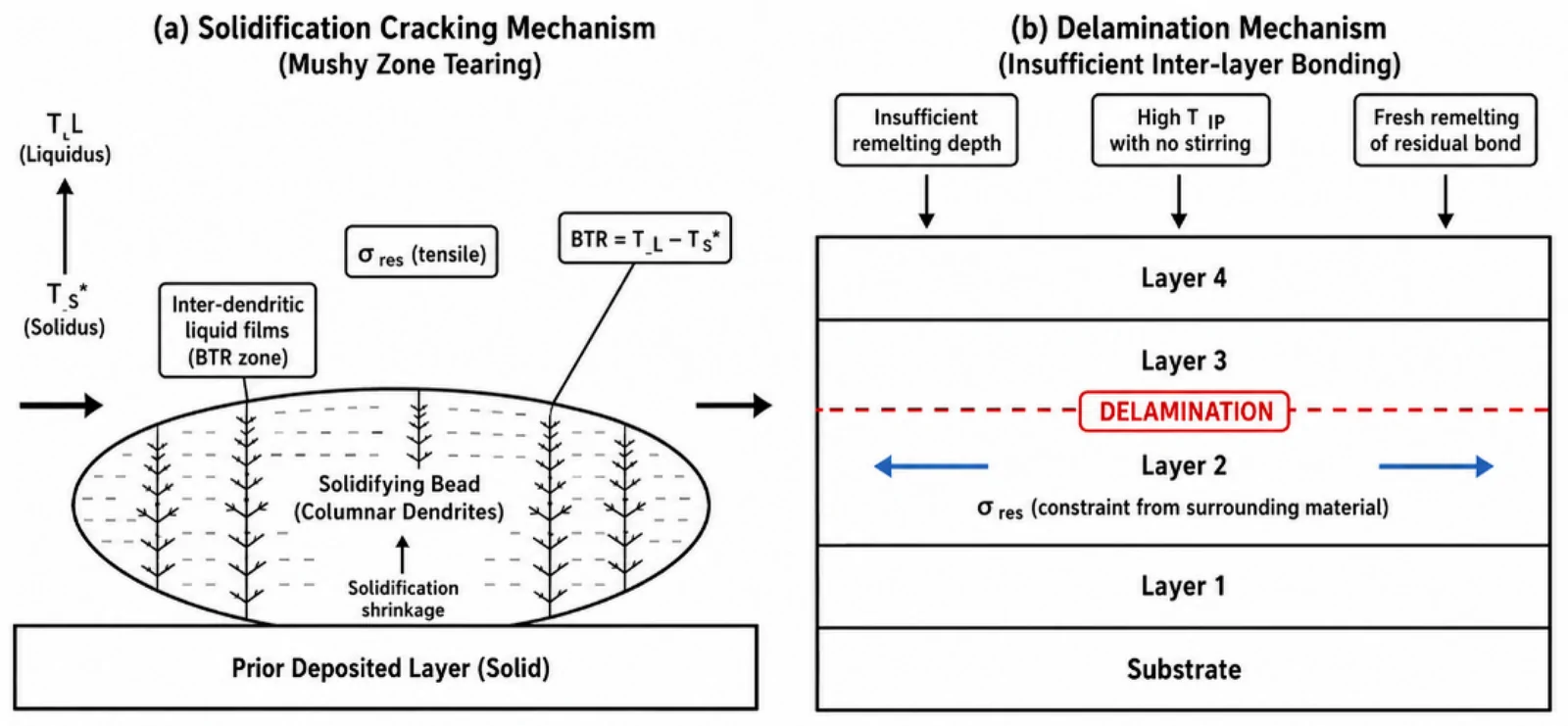
Conceptual schematic, generated with ChatGPT (GPT-5) from author-specified content, illustrating key defect formation mechanisms in WAAM: (**a**) solidification cracking driven by tensile residual stress acting across the brittleness temperature range, (*BTR* = *T_L_* − Ts*), where interdendritic liquid films remain vulnerable to tearing; and (**b**) delamination at the interlayer interface when residual stress exceeds the bonding strength of a partially remelted interface. No numerical data or graphical content were reproduced, adapted, digitized, or collected from previously published sources.

This subsection summarizes the main defect mechanisms and governing relationships in WAAM. Figure 18 shows two important defects related to thermal stress and inter-layer bonding. Figure 18a shows solidification cracking as a mushy-zone tearing mechanism. During solidification, liquid films remain between growing dendrites within the brittleness temperature range (BTR). This region is weak because it is not fully solidified. When tensile residual stress acts across this semi-solid zone, the liquid film can rupture and form a crack. This problem is more serious in alloys with a wide solidification range, strong solute segregation, and poor resistance to thermal contraction [16].

Figure 18b shows delamination caused by weak inter-layer bonding. In WAAM, each new layer must partially remelt the previous layer to form a sound metallurgical bond. If remelting is insufficient, or if oxidation and unsuitable inter-pass temperature affect the interface, a weak bonding zone may remain. During cooling, tensile residual stress develops due to constrained thermal contraction. If this stress exceeds the inter-layer bond strength, separation occurs along the layer interface. Therefore, delamination is both an inter-layer bonding defect and a thermomechanical failure mode.

As summarized in Table 5, WAAM defects include porosity, solidification cracking, residual stress, delamination, columnar grain coarsening, and geometric deviation. These defects are controlled by heat input, melt-pool stability, inter-pass temperature, solidification behavior, shielding quality, and deposition path. Porosity is mainly linked to hydrogen rejection and gas entrapment. Solidification cracking is caused by tensile stress acting on semi-solid interdendritic liquid films. Residual stress forms due to constrained thermal contraction. Delamination occurs when the inter-layer bond is too weak to resist cooling stress. Columnar grain coarsening is related to epitaxial growth along the build direction. Geometric deviation is linked to bead instability and heat accumulation during multilayer deposition.

Table 5 connects these defects with the main equations used in WAAM analysis. The heat input equation explains how the supplied thermal energy controls melt-pool size, cooling rate, remelting depth, and residual stress. Grain-growth and Hall–Petch relationships explain why repeated heating can coarsen grains and why grain refinement can improve strength. Hydrogen diffusion and solubility relationships help explain porosity formation. The thermal strain equation explains residual stress and distortion. The *G*/*R* ratio and dendrite arm-spacing relationships explain solidification mode, dendrite refinement, segregation, and grain morphology. Figure 18 and Table 5 and Table 6 show that WAAM defects are controlled by combined thermal, metallurgical, and mechanical factors, not by one process parameter alone.

## 8. Quantitative and Alloy-Specific Synthesis of WAAM Performance

The preceding sections show that WAAM performance is governed by coupled process, thermal, microstructural, and defect-related mechanisms. A quantitative synthesis is therefore useful, but it must be interpreted cautiously because reported values across the literature differ in alloy condition, build geometry, testing direction, measurement location, and post-processing state.

### 8.1. Representative Quantitative Trends Across the Literature

With this caveat in mind, Table 6 compiles quantitative results that could be traced to the original publications according to the criteria in Section 2.3. For each entry, the controlled variable and the condition to which each value refers are stated, so that values are compared within, not across, studies.

Although the entries in Table 6 cannot be pooled statistically, three observations are consistent with the mechanisms discussed in Section 4, Section 5, Section 6 and Section 7. First, when the heat input or the inter-pass temperature is varied within a single study, the defect or property response is non-monotonic, with optima at intermediate conditions: porosity passed through a minimum at an intermediate hot-wire current [44], and tensile strength peaked at an intermediate inter-pass temperature before the wall collapsed at the highest one [63]. Second, the transfer-mode variant changes the heat input at nominally similar deposition conditions, as shown for CMT variants in martensitic stainless steel [51]. Third, inter-layer or post-deposition treatments act on features that the arc process alone does not remove, such as the coarse as-deposited grain structure of aluminum walls [74] or Laves phase retained near a dissimilar interface [75].

Build-height gradients in grain size and hardness follow from the same thermal history. As a wall grows, heat accumulates and the effective temperature gradient at the solidification front decreases (Section 6.2), so grain size tends to increase and hardness to fluctuate along the build direction, with bands corresponding to regions reheated above and below the relevant transformation or precipitation temperatures [40,70]. The magnitude of these gradients is alloy- and geometry-specific and should be quantified by layer-resolved measurements within a given build rather than inferred from values compiled across studies. More generally, the underlying mechanisms are strongly alloy-dependent. Although the heat-source and solidification principles discussed above are common to all WAAM processes, their microstructural consequences differ considerably among alloy families because phase transformation behavior, thermal conductivity, solidification range, hydrogen solubility, and precipitation kinetics vary significantly.

### 8.2. Ferrous and Stainless Steels

In mild steels and low-alloy steels, WAAM deposits may exhibit columnar prior austenite grains in as-solidified regions, which subsequently transform into ferrite–pearlite, bainite, or martensite depending on alloy composition and cooling rate. The continuous cooling transformation (CCT) diagram governs the transformation product for a given cooling rate. Dickens et al. [40] documented a wall microstructure comprising large equiaxed ferrite (α) and pearlite (α + Fe_3_C) with grain sizes of approximately 60 µm in reheated mid-wall regions. In unreheated top-layer regions, columnar austenite grains transformed to coarser Widmanstätten ferrite colonies, with reported dimensions of approximately 600 µm × 1000 µm. The practical implication is that the uppermost layers of a WAAM steel component may exhibit lower toughness and ductility than the thermally cycled bulk, requiring consideration of post-process heat treatment, surface machining, or location-specific property qualification in toughness-critical applications [12,32,40].

For 316L stainless steel WAAM, solidification is commonly described as austenitic or austenite-dominant depending on composition and thermal history. The deposit may exhibit columnar austenite grains with residual ferrite stringers along grain boundaries, produced by segregation of ferrite-stabilizing elements such as Cr and Mo during solidification. Repeated reheating cycles in mid-build regions can partially homogenize this segregation pattern, but may also promote secondary-phase precipitation at grain boundaries under certain thermal exposure conditions, which is a concern for corrosion resistance and embrittlement in elevated-temperature service [32,78,79].

### 8.3. Aluminum and Titanium Alloys

Aluminum alloys present the most complex suites of WAAM microstructural challenges owing to: (i) the high hydrogen solubility ratio at the melting point, which drives porosity; (ii) wide solidification temperature ranges in high-solute alloys, which increase hot-cracking susceptibility; (iii) sensitivity of strengthening precipitates to dissolution or over-aging during reheating; (iv) high thermal conductivity, which modifies the thermal gradient at the solidification front; and (v) the stable refractory oxide layer that rapidly re-forms on exposed aluminum surfaces and can reduce inter-layer bonding unless disrupted by arc action.

The microstructure of WAAM aluminum alloys is commonly characterized by columnar grains with cellular or columnar-dendritic substructures. The inter-dendritic eutectic constituents depend on alloy series: β-Al_3_Mg_2_ in 5xxx alloys, θ-Al_2_Cu in 2xxx alloys, and eutectic Si in 4xxx alloys [16]. The 4xxx series silicon-bearing alloys often show better WAAM processability than many higher-solute aluminum alloys because their narrower solidification temperature range reduces the brittleness temperature range [16]. In GMAW-based deposition of common aluminum filler alloys, the 4000-series wires gave better bead width, porosity, strength, and defect sensitivity than ER1100 and ER5356, and ER5356 showed the highest porosity [77]. With variable-polarity CMT, Zhang et al. [80] obtained equiaxed grains of 20.6–28.5 µm with random orientation in Al-6Mg deposits, whereas other arc modes produced larger columnar grains; the tensile strength reached 333 MPa but was 8–27% anisotropic because of micropores in the interlayer regions. In AlMg5Mn deposited with the CMT, CMT Advanced, and CMT Pulse Advanced modes, Gierth et al. [81] controlled the layer geometry and kept the porosity area fraction below 1% by adjusting the energy input per unit length, which also changed the tensile strength and hardness.

Vimal et al. [70] reviewed WAAM of aluminum alloys and, consistent with other reviews [7,14,82], identified six primary challenges: (i) porosity from hydrogen rejection; (ii) tensile residual stress; (iii) cracks and deformation when *σ_res_* > *σ_UTS_*; (iv) delamination; (v) difficulty in processing 7xxx series alloys by single-wire WAAM; and (vi) turbulent melt pool development in 7xxx and 6xxx series due to high vapor pressure of volatile alloying elements such as Zn and Mg. For the 7xxx and 6xxx alloys specifically, a turbulent melt pool promotes oxide film entrainment and bifilm defects, which can be difficult to detect using conventional ultrasonic inspection and can considerably reduce ductility and fatigue life [70]. The sensitivity of fatigue life to pore geometry has been quantified for additively manufactured Ti-6Al-4V, for which finite element analysis gave stress concentration factors of 2.08 for internal spherical pores and 2.5 for internal oblate pores and identified subsurface pores as the most critical [83].

Ti-6Al-4V is the most studied titanium alloy in WAAM because of its widespread aerospace applications [46,84,85]. Using PAW-based WAAM in an argon atmosphere, Artaza et al. [46] deposited Ti-6Al-4V walls at 2 kg/h with oxygen levels below 100 ppm, and the annealed material met the AMS 4928 yield and tensile strength requirements whether heat treatment was performed in vacuum, air, or argon. In WAAM, Ti-6Al-4V solidifies as body-centered cubic β, forming columnar β grains with a strong <001> texture parallel to the build direction. These columnar β grains are stable above the β transus, approximately 995 °C, and transform on cooling to α + β microstructures, including lamellar α colonies or Widmanstätten α within the β matrix, with the specific morphology governed by local cooling rate. In Jominy end-quench experiments on Ti-6Al-4V, cooling rates above 410 °C/s produced a fully martensitic microstructure; a massive transformation occurred between 410 and 20 °C/s, and this was progressively replaced by diffusion-controlled Widmanstätten α as the cooling rate decreased further [68]; the rates prevailing in a given WAAM deposit depend on the heat input and on the reheating history [13,85].

The repeated thermal cycling of WAAM reheats prior β columnar grains below the β transus, driving solid-state transformation without re-solidification. Progressive decomposition of as-formed martensite α’ to equilibrium α + β, and coarsening of α laths with increasing thermal exposure, produce the characteristic banded microstructure of WAAM Ti-6Al-4V. This banding can include alternating regions of finer α morphologies in zones that experienced higher thermal exposure and coarser lamellar α colonies in regions subjected to lower thermal exposure. Because the mechanical properties of Ti-6Al-4V are strongly dependent on α morphology and prior-β grain structure, these microstructural bands produce property gradients that remain a significant challenge for certification of WAAM titanium components for aerospace applications.

### 8.4. Nickel-Based Superalloys and Cross-Alloy Performance Comparison

Nickel-based superalloys, including Inconel 625, Inconel 718, and Hastelloy-type alloys, are challenging materials for WAAM because of their high alloying content, strong segregation tendency, potential formation of Laves and other secondary phases, high residual stress generation, and sluggish recrystallization kinetics that can limit grain refinement. In WAAM of Inconel 625 and Inconel 718, columnar γ grains with a strong ⟨001⟩ texture are commonly reported, and Laves phase enriched in Nb (and Mo in Inconel 625) can form in interdendritic regions because of solidification segregation [75,76]. In GTAW-based deposition of Inconel 625, Wang et al. [76] observed cellular grains near the substrate, upward-oriented columnar dendrites in the layer bands, and a transition to equiaxed grains in the top region; Nb and Mo segregation strengthened with deposit height, the primary dendrite arm spacing correlated with the Laves content, and the near-substrate region had the highest microhardness and tensile strength.

The Laves phase in nickel-superalloy WAAM is particularly detrimental because it can raise local hardness, reduce toughness, and serve as a crack-initiation site under thermal or mechanical cycling. Post-WAAM solution treatment above the Laves solvus can dissolve this phase and redistribute segregating elements more homogeneously, but such high-temperature treatments must be balanced against undesirable grain growth. Hasani et al. [75] used elastic-field models, informed by neutron diffraction and nanohardness data collected across a WAAM IN718/S275 interface, to interpret how heat treatment changes dislocation mobility and the Peierls–Nabarro stress, and reported that Laves phase persisted near the interface even after solution treatment at 1080 °C for 1 h.

**Figure 19:**
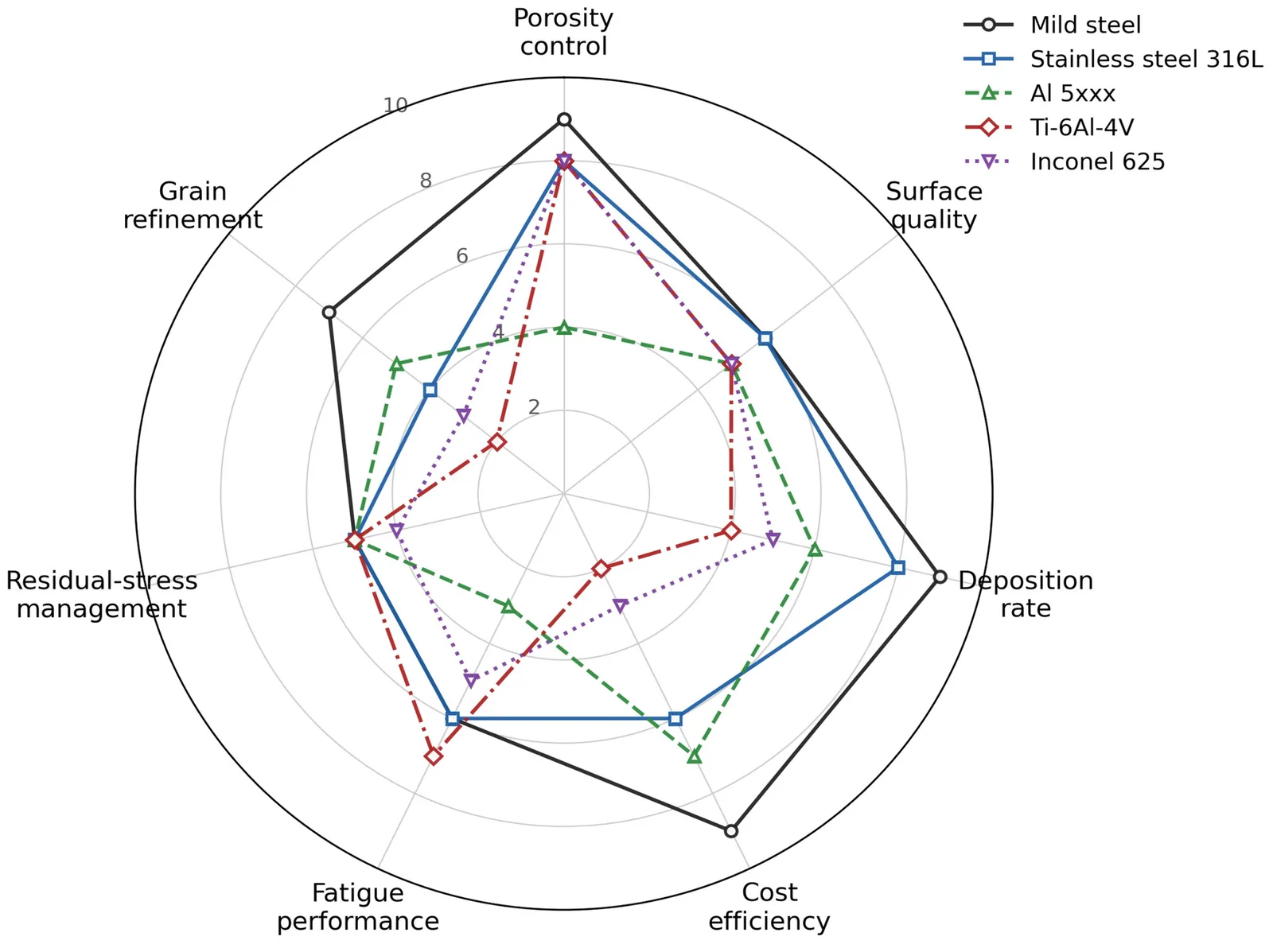
Qualitative comparison of WAAM processability and performance criteria for five alloy families. Ratings are author-assigned ordinal assessments on a 1–10 scale using the anchor descriptors in Table 7, with equal weighting of criteria; they are not measured, extracted, or normalized literature values. Chart drawn with Python (Matplotlib 3.10) on a linear radial scale.

Figure 19 presents a qualitative comparison of five alloy families across seven processing and performance criteria. The ratings are expert ordinal assessments by the authors, not measured, extracted, or normalized values, and the figure is intended as a qualitative comparison only.

The ratings were assigned on a 1–10 ordinal scale using the anchor descriptors in Table 7, in which 1–3 denotes a criterion that is a principal limitation for WAAM of the alloy family, 4–6 a criterion that can be managed with established process controls, and 7–10 a criterion that is favorable under typical WAAM conditions. All seven criteria were weighted equally, no aggregate score was calculated, and no literature data were converted to the scale. The ratings reflect the authors’ synthesis of the reviewed literature [7,14,31,32,34,70,82] and should be regarded as indicative.

In the ratings shown, mild steel is rated most favorably overall, particularly for deposition rate, cost efficiency, and grain refinement. The aluminum 5xxx ratings are limited mainly by porosity control and fatigue performance, the Ti-6Al-4V ratings by cost efficiency, grain refinement, and deposition rate, and the Inconel 625 ratings by cost efficiency, grain refinement, and residual-stress management.

Figure 19 shows that no alloy family is ideal in all categories. WAAM performance depends strongly on the balance between deposition efficiency, defect control, microstructure, mechanical performance, and cost.

### 8.5. Comparison with Multi-Pass Weld Metal

A systematic comparison of WAAM deposits with multi-pass weld metal of the same alloy, process, and heat input would show directly which characteristics are distinctive to WAAM. Matched datasets of this kind, with the same filler wire, process, heat input, and test orientation, are rarely reported; most WAAM studies compare their results with wrought material [21,78]. The mechanisms discussed in Section 6 and Section 7 nevertheless allow the characteristics to be classified. Shared with multi-pass weld metal are the dendritic solidification structure and microsegregation governed by *G*, *R*, and the solidification range; *δ*-ferrite and its transformation products in austenitic stainless steels [78]; tempering and re-transformation of earlier passes in steels; and hydrogen porosity in aluminum alloys. Distinctive to WAAM, or more pronounced in it, are columnar grains and crystallographic texture aligned with the build direction over the full height of the component [75], and the resulting anisotropy; build-height gradients in grain size, segregation, and hardness caused by the evolving heat-extraction path [66,76]; the absence of dilution, so that the deposit chemistry equals the wire chemistry and cannot be adjusted by a parent metal; and residual stress fields that develop in a free-standing wall and redistribute when it is released from the substrate [25,26]. Establishing these differences quantitatively requires matched WAAM and multi-pass weld specimens, which remains a research need.

### 8.6. WAAM-Specific Feedstock

Most WAAM wires are conventional welding consumables, whose compositions were designed to give sound joints after dilution with the parent metal in one or a few passes [21]. In WAAM, the wire alone forms the component; there is no dilution, and the deposit is reheated many times, so compositions that are adequate for joining can be unsuitable for bulk deposition. Feedstock tailored for WAAM has followed three routes. For aluminum alloys, wires micro-alloyed with Sc and Zr form Al_3_(Sc,Zr) particles that nucleate equiaxed grains and reduce hot-cracking susceptibility, and tailored fillers have enabled the deposition of high-strength Al–Zn–Mg–Cu compositions that crack when deposited with standard wires [21,86]. For titanium and nickel alloys, modified chemistries, such as β-stabilizer additions to refine prior-β grains in Ti-6Al-4V and reduced Nb in 718-type wires to limit Laves formation, and impurity limits stricter than those for welding consumables have been proposed [21]. For duplex stainless steels, wires with adjusted N, Ni, and Mn contents are used to maintain the ferrite–austenite balance under repeated reheating [21]. These modifications address the WAAM-specific conditions discussed in Section 6 and Section 7: the absence of dilution, the columnar growth promoted by directional heat extraction, and the cumulative reheating of the deposit.

## 9. Industrial Readiness, Advantages, Limitations and Qualification Challenges

The process and metallurgical characteristics discussed above explain why WAAM is attractive for large-scale metallic component production. At the same time, they also explain why industrial qualification remains challenging. WAAM offers clear advantages in deposition rate, material utilization, component scale and cost, but these benefits must be balanced against surface finish limitations, microstructural heterogeneity, residual stress, porosity, process complexity and certification requirements.

### 9.1. Industrial Advantages and Persistent Technical Limitations

The technical trends discussed in Section 3, Section 4, Section 5, Section 6, Section 7 and Section 8 explain why WAAM is attractive for industrial manufacturing, but they also show why qualification remains challenging.

- **Near-net-shape capability:** WAAM can reduce buy-to-fly (BTF) ratios to approximately 1.5:1 to 3:1 for aerospace titanium components, compared with 12:1 to 20:1 by conventional machining [1,14].
- **Scalability:** WAAM can fabricate components ranging from sub-kilogram prototypes to multi-tonne structural parts several meters in dimension, a scale that remains difficult and economically impractical for most powder-based AM processes.
- **High deposition rate**: WAAM can achieve deposition rates of approximately 1–10 kg/h, typically 2–4 kg/h for GMAW-WAAM, which is appreciably higher than powder-bed fusion processes.
- **Low capital and operating costs**: WAAM uses standard industrial welding equipment, widely available wire feedstock, and established maintenance infrastructure.
- **Near-100% material utilization**: Wire feedstock is fully consumed, unlike powder processes requiring powder recycling and contamination management.
- **Multi-axis deposition flexibility**: Multi-axis robotic systems enable deposition on non-planar surfaces and complex fixture geometries, although the achievable geometric resolution remains lower than powder-bed processes.
- **Broad feedstock availability**: Standard welding wires in steel, aluminum, titanium, nickel, copper, and intermetallic alloys are commercially available at low cost.
- **Established metallurgical knowledge base**: The extensive body of welding metallurgy science provides a direct foundation for WAAM process development and defect understanding.

Despite these advantages, WAAM also presents several persistent challenges that must be addressed before wider industrial qualification and certification.

- **Surface finish**: Bead-on-bead deposition produces surface waviness, commonly with Ra values of approximately 10–50 µm, requiring post-deposition machining in most structural applications.
- **Microstructural heterogeneity:** The layered thermal history produces non-uniform microstructures from base to top, including columnar grain continuity and property anisotropy that complicate structural design and certification.
- **Residual stress and distortion**: Cyclic thermal loading can generate residual stresses approaching yield strength, driving distortion and, in severe cases, cracking or delamination.
- **Porosity**: Porosity is particularly severe in aluminum alloys, driven by the high liquid-to-solid hydrogen solubility ratio and compounded by turbulent melt pool dynamics.
- **Process parameter complexity**: The large parameter space requires systematic design of experiments, response surface methods, or data-driven optimization to achieve consistent part quality.
- **Certification gap**: The lack of mature, process-specific qualification standards for WAAM components remains the primary barrier to adoption in regulated industries.

Taken together, these advantages and disadvantages show that WAAM is not limited by deposition capability alone, but by the ability to control the process–structure–property relationship across large volumes. This is why thermal management, inter-pass control, hybrid post-processing, and qualification standards remain central to the transition from laboratory-scale deposition to certified industrial production.

### 9.2. Technology Readiness and Certification Barriers

After considering WAAM’s technical advantages and limitations, the next question is whether the process is mature enough for reliable industrial deployment [87]. The Technology Readiness Level (TRL) framework, originally developed by NASA and subsequently adopted by organizations such as the European Space Agency, the US Department of Defense, and the European Commission for Horizon research programs, provides a standardized nine-level scale for assessing the maturity of a technology from basic research principles (TRL 1) to fully deployed, flight-proven systems (TRL 9) [87]. Applying this framework to WAAM enables a structured assessment of where the technology currently stands and which technical, industrial, and regulatory barriers must be overcome to advance toward routine deployment.

**Figure 20:**
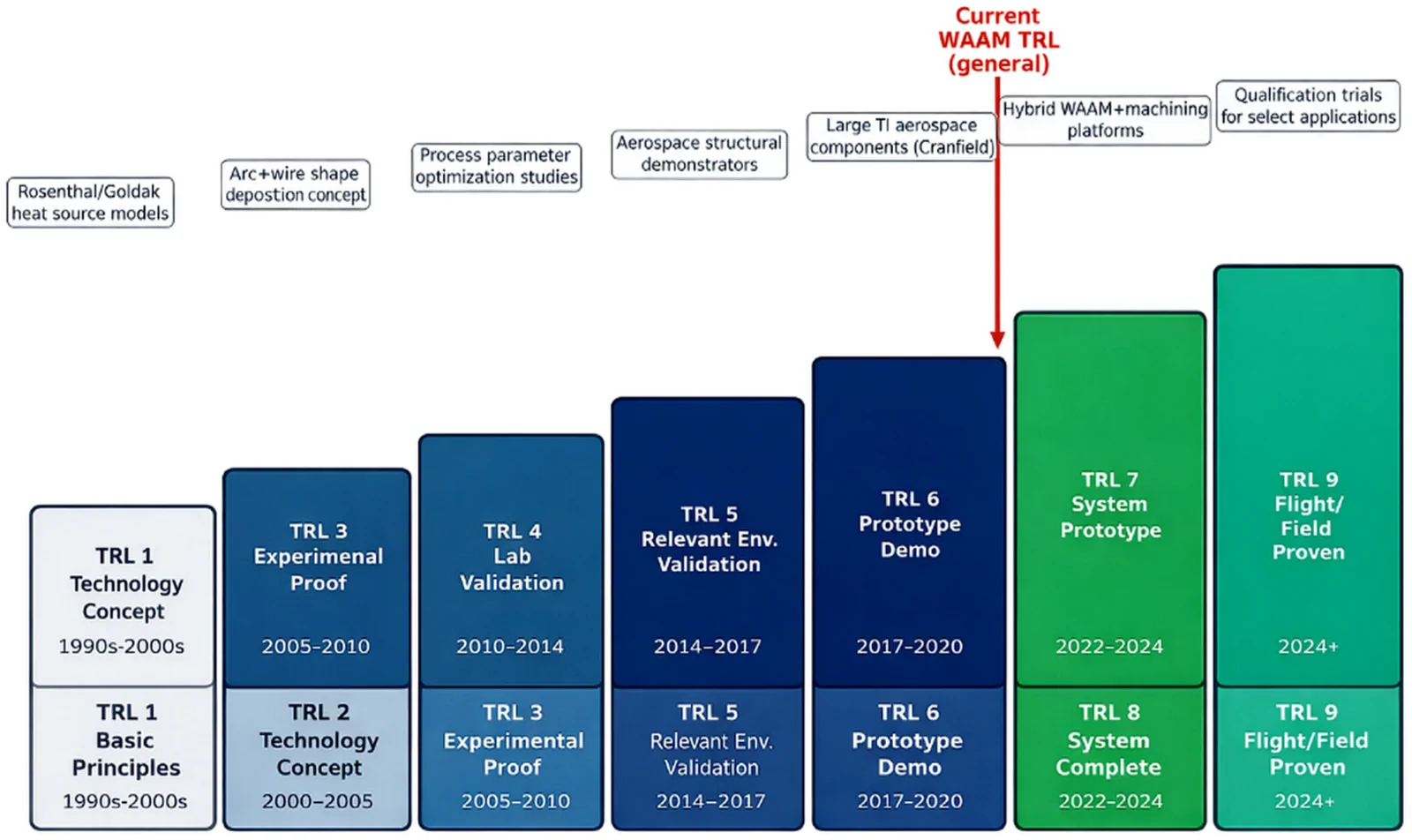
Technology Readiness Level (TRL) progression for WAAM from basic research principles to current industrial deployment. WAAM maturity is application-specific, with selected steel and titanium applications approaching TRL 6–7, while many aluminum and nickel-based superalloy applications remain closer to TRL 5–6. Conceptual schematic generated with ChatGPT (GPT-5) from author-specified content; TRL definitions follow Mankins [87], and no numerical data were collected, digitized, or adapted from a published source.

Figure 20 presents the TRL progression for WAAM from its conceptual origins in Baker’s 1925 patent [8] to the current state of the art. Based on the literature reviewed and selected industrial case studies, WAAM may be considered to have reached approximately TRL 6–7 for selected steel and titanium alloy applications, whereas many aluminum and nickel-based superalloy applications remain closer to TRL 5–6. Specific applications, such as WAAM-fabricated titanium brackets, structural frames, and selected large-scale demonstrator components reported by Cranfield University and aerospace industrial partners, may approach higher TRL levels, but these examples should be interpreted as application-specific rather than representative of all WAAM materials and geometries. While TRL provides a useful maturity indicator, certification readiness depends on whether WAAM components can meet reproducible property allowables, defect acceptance criteria, and inspection requirements.

The TRL assessment presented here is intended as a qualitative, application-dependent maturity map rather than a fixed universal rating; TRL depends on the material, component geometry, qualification route, and end-use sector [87].

The primary barrier preventing the broader transition of WAAM from demonstrated capability toward routine qualification in regulated industries, particularly aerospace, nuclear, and pressure-containing applications, is not only technical performance but also regulatory qualification. Current aviation certification frameworks, such as FAR Part 25 and CS-25, were developed primarily for conventionally manufactured metallic components and do not yet provide WAAM-specific qualification pathways. The key certification challenges are:

- **Microstructural heterogeneity and anisotropy**: Certification requires demonstration of consistent properties meeting minimum allowables across the relevant part volume. WAAM’s inherent base-to-top microstructural gradient and build-direction anisotropy make this demonstration difficult without extensive sampling, process control, and location-specific property assessment.
- **Porosity detection and acceptance criteria** [88,89]: Non-destructive evaluation (NDE) of WAAM components for porosity using conventional ultrasonic testing can be complicated by anisotropic grain structures, which scatter ultrasound and reduce flaw-detection sensitivity. The smallest detectable defect depends on grain size, anisotropy, surface condition, and section thickness; phased-array ultrasonic testing has detected defects of 0.6–1 mm in aluminum WAAM components, whereas X-ray computed tomography, although capable of finer resolution, is too slow for real-time or in-line assessment [89].
- **Residual stress qualification**: No universally adopted production-scale method currently exists for qualifying WAAM components against residual-stress-related failure modes. Neutron diffraction and synchrotron X-ray diffraction can map residual stress in three dimensions, but they are not accessible for routine production inspection [90,91].
- **Fatigue design allowables**: Fatigue allowables for WAAM, analogous to established allowables for conventional alloys, remain insufficient for many alloy/process combinations. The sensitivity of WAAM fatigue performance to microstructural heterogeneity, surface waviness, porosity, and residual stress requires systematic, statistically robust S-N curve development before routine certification in safety-critical structural applications.

International standardization bodies are actively developing and updating AM and DED-related standards. ISO/ASTM 52900:2021 remains the current terminology standard for additive manufacturing, while ASTM F3187-16(2023) provides guidance for directed energy deposition of metals, including systems using laser beam, electron beam, and arc/plasma-based energy sources [92,93]. These standards provide important terminology and process-guidance frameworks, but they do not by themselves establish complete material allowables, fatigue databases, or component-specific qualification routes for all WAAM applications. Ongoing work by the ASTM F42 and ISO/TC 261 committees, and by aerospace materials committees, is therefore essential, but progress in standardization remains slower than the pace of WAAM process development.

### 9.3. Industrial Case Studies and Deployment Status

Despite the certification gap, WAAM has achieved meaningful industrial deployment in several application domains. In aerospace, Cranfield University’s WAAMMat (Wire + Arc Additive Manufacturing Materials) program demonstrated multi-meter titanium spars and wing ribs with buy-to-fly ratios below 3:1, validated against conventional machined counterparts [1,14,84]. Airbus and Spirit AeroSystems have reported exploratory programs for WAAM titanium brackets. In defense, BAE Systems and Lockheed Martin have evaluated WAAM for the repair and fabrication of naval and aerospace components [84].

In the energy sector, where qualification routes may be less restrictive than aerospace depending on component criticality, WAAM has been explored for the manufacture and repair of large valve bodies, impellers, propellers, and pressure-vessel-related components in oil and gas applications [7,31]. The marine sector has also demonstrated one of the most visible WAAM qualification examples: the WAAMpeller project, a nickel–aluminum bronze ship propeller fabricated by Wire Arc Additive Manufacturing and unveiled in 2017 after testing verified by Bureau Veritas [94]. These examples indicate that WAAM is already viable for selected high-value and large-scale components, but broader adoption still depends on repeatable process control, defect acceptance criteria, property allowables, and application-specific certification routes.

## 10. Discussion: Critical Contradictions, Hybrid Strategies and Future Research Priorities

The preceding sections establish the process, metallurgical, defect, alloy and industrial contexts of WAAM. The scientific value of interpreting WAAM through traditional welding principles lies not in treating WAAM as repeated welding, but in identifying where the welding framework remains valid and where it breaks down under layer-wise thermal accumulation. This distinction provides the basis for discussing unresolved contradictions, hybrid process strategies and future research priorities.

### 10.1. Critical Contradictions and Process-Microstructure Interpretation

A careful review should not only synthesize areas of agreement but also identify genuine scientific disagreements that define the most productive frontiers for future research. Several important contradictions exist in the WAAM literature.

**Travel speed and solidification structure**: Ou et al. [65] predicted that a faster travel speed increases the cooling rate *G*·*R* and decreases *G*/*R*. The first effect refines the dendrite arm spacing (Equation (20)), whereas the second shifts the solidification morphology toward columnar-dendritic or equiaxed growth (Equations (18) and (19)). Apparent disagreements on whether faster travel coarsens or refines WAAM microstructures usually arise because studies report different length scales (primary dendrite arm spacing, columnar grain width, or equiaxed grain size) and because travel speed is rarely varied at constant arc power and deposition rate: reducing the bead cross-section at a higher speed also changes the number of layers, the heat accumulation, and the reheating history of the wall. A meaningful comparison therefore requires that the measured length scale and the co-varied parameters be stated explicitly.

**Inter-pass temperature and defect formation**: Geng et al. [60] reported that lower inter-pass temperature reduces heat accumulation and improves dimensional accuracy in aluminum WAAM. Conversely, an excessively low inter-pass temperature reduces the effective preheat of the prior layer surface and can increase the risk of insufficient remelting and delamination [70]. Rios et al. [61] further showed that the inter-pass temperature enters the prediction of deposit geometry. These findings indicate that inter-pass temperature is a critical but difficult-to-standardize variable, and that a single optimal value cannot be defined without specifying alloy system, deposit geometry, process variant, and environmental conditions.

**CMT variants versus pulsed GMAW**: Galeazzi et al. [51] found that CMT Advanced and CMT Pulse lay at opposite extremes of heat input among the CMT variants for AISI 420 martensitic stainless-steel walls, whereas Luo et al. [53] found that a pulsed arc outperformed a non-pulsed arc in aluminum WAAM because of its higher droplet-transfer frequency and deposition efficiency. These studies differ in alloy, thermal conductivity, transfer mode, and evaluation criteria (wall geometry and heat input versus droplet transfer and deposition efficiency), so they do not constitute a direct contradiction; they do show that the ranking of CMT and pulsed GMAW variants is material- and criterion-specific and must be established for the alloy and component of interest.

**Porosity and heat input optima**: While many studies agree on the non-monotonic porosity–heat-input relationship, the location of the optimum differs considerably among alloy systems and process variants. Fu et al. [44] identified a porosity minimum at a hot-wire current of 100 A in hot-wire arc additive manufacturing of aluminum, while Haselhuhn et al. [77] found that, under common GMAW printing conditions, porosity remained below 2% for all tested aluminum filler alloys but was highest for ER5356, with the 4000-series alloys performing best. These discrepancies confirm that porosity management protocols are alloy-specific and cannot be transferred across systems without experimental re-validation.

The most important conceptual unification emerging from this review is the central role of the *G*/*R* ratio as the governing parameter linking WAAM process conditions to microstructural outcome. As demonstrated through Equations (18)–(20), *G*/*R* controls the solidification mode ranging from planar to equiaxed-dendritic, while *G*·*R* controls cooling rate and dendrite arm spacing. Both parameters vary continuously in three dimensions around the melt pool, meaning that a single deposited WAAM bead can contain a gradient of microstructures from planar or cellular regions near the fusion boundary to columnar-dendritic regions in the melt-pool interior, as represented schematically in Figure 15.

The transition between columnar and equiaxed solidification is controlled by the local thermal gradient *G*, solidification rate *R*, cooling rate *G*·*R*, and the degree of constitutional undercooling ahead of the solidification front. Higher *G*/*R* generally favors planar or columnar growth, whereas lower *G*/*R* combined with sufficient undercooling and available nucleation sites can promote equiaxed growth [33,71,72].

Cumulative effects of repeated deposition on *G* and *R* are not unique to WAAM; they also occur in multi-pass welding. The difference lies in the geometry and boundary conditions. In a multi-pass joint, each bead solidifies against the groove faces and the previous beads, so the direction of *G* changes with bead placement and the columnar grains change orientation from pass to pass, while the parent plates maintain a lateral heat sink throughout. In a WAAM wall, the fusion boundary advances continuously upward, *G* remains aligned with the build direction, and its magnitude decreases as the heat-extraction path lengthens [66], so grains can grow epitaxially through many layers with a common orientation. As build height increases, the effective heat sink distance from the melt pool to the external boundary increases, often reducing *G* at the solidification front and shifting the operating point on the solidification map toward a different dendritic regime. This progressive reduction in *G* with build height is the physical basis for the commonly observed increase in grain size from the base to the top of WAAM builds.

The practical implication for WAAM process engineers is that achieving microstructural homogeneity across build height requires active compensation for this evolving thermal boundary condition. Possible strategies include decreasing *H* with build height through adaptive process control, increasing substrate or inter-pass cooling, or introducing external perturbations to the melt pool, such as arc oscillation, ultrasonic assistance, magnetic-field assistance, grain refiners, or inter-layer deformation, to promote equiaxed nucleation or disrupt columnar growth. These approaches are active research areas, but their robustness across alloy systems, build geometries, and industrial-scale production remains insufficiently established.

A recurring and practically important finding across the reviewed literature is the non-monotonic relationship between heat input and porosity in aluminum WAAM. At low heat input, insufficient preheating of the wire and substrate may leave hydrogen-bearing surface contamination intact, increasing the hydrogen source term in Sieverts’ law. At high heat input, the enlarged melt pool increases Marangoni-driven flow, extends the solidification time, and promotes oxide-film entrainment, all of which can increase pore entrapment. An intermediate optimum therefore exists, such as the hot-wire current of 100 A reported by Fu et al. [44], at which preheating and melt-pool size are balanced.

This optimal window is alloy-specific, feedstock-condition-specific, and geometry-specific, which explains the persistent difficulty of transferring WAAM porosity solutions from one application to another. A physics-informed approach to porosity management must address all three hydrogen source categories: (i) surface contamination, controlled by wire and substrate cleaning protocols; (ii) shielding gas moisture, controlled by gas quality, line dryness, and purging; and (iii) melt pool hydrodynamics, controlled by process parameters and bead geometry optimization.

The analysis in Section 6.4 shows that the thermal strain driving residual stress is *α_CTE_*·*ΔT*, so *E*·*α_CTE_* alone does not rank alloys; the temperature change must be included, and the tendency toward plastic flow and distortion is best expressed as *E*·*α_CTE_*·*ΔT*/*σ_y_*. For a deposit that cools from the temperature at which it begins to support stress to the inter-layer temperature, the upper bound of *ΔT* is *T_S_* − *T_IP_*, where *T_S_* is the solidus temperature, and this bound differs strongly between alloys. For an aluminum alloy with *T_S_* ≈ 580 °C and *T_IP_* ≈ 100 °C, *ΔT* ≲ 480 K and *E*·*α_CTE_*·*ΔT* ≈ 0.8 GPa (*E* ≈ 70 GPa, *α_CTE_* ≈ 23 × 10^−6^ K^−1^); for Ti-6Al-4V with *T_S_* ≈ 1600 °C and *T_IP_* ≈ 200 °C, *ΔT* ≲ 1400 K and *E*·*α_CTE_*·*ΔT* ≈ 1.4 GPa (*E* ≈ 110 GPa, *α_CTE_* ≈ 9 × 10^−6^ K^−1^). Both values exceed the room-temperature yield strength, so the residual stress is limited by the temperature-dependent yield strength rather than by the elastic estimate; because *E* and *σ_y_* fall steeply as the solidus is approached, the effective ΔT over which stress accumulates is smaller than *T_S_* − *T_IP_*. For aluminum, the low yield strength of the as-deposited material means that thermal strains exceed the elastic limit readily, as shown by longitudinal tensile residual stresses reaching the flow strength in 2319 aluminum walls [25], whereas in nickel superalloys the high *E*·*α_CTE_* combined with high elevated-temperature strength is expected to produce large residual stresses. In both cases residual stresses can approach the yield strength, driving distortion and, in susceptible alloys, cracking. This thermomechanical constraint cannot always be eliminated by process-parameter adjustment alone and provides a rationale for hybrid WAAM approaches incorporating inter-layer rolling, peening, friction stir processing, or other mechanical treatments that introduce plastic strain, refine grains, and partially counteract the tensile residual-stress field.

### 10.2. Hybrid WAAM Strategies: Deformation, Machining, and Low-Heat-Input Transfer

Hybrid WAAM strategies couple arc deposition with a second, non-fusion operation that acts on the deposit between layers or after deposition. They can be grouped by the mechanism through which they modify the as-deposited state: (i) mechanical deformation of each layer or of the completed wall (inter-layer rolling, peening, and friction stir processing), which introduces plastic strain that disrupts epitaxial columnar growth on reheating and redistributes residual stress; (ii) machining between or after layers, which restores geometric accuracy but does not change the microstructure; and (iii) thermal or field-assisted control of solidification, such as forced inter-pass cooling, arc oscillation, and ultrasonic or magnetic assistance, which acts on the melt pool rather than on the solid deposit. Table 8 compares the principal deformation- and machining-based strategies on a common basis.

Rolling, peening, and magnetic fields are also used in conventional welding, but with different purposes, timing, and effects. In welding, they are usually applied once, after the joint is complete, to reduce distortion or residual stress or to improve the fatigue strength of the weld toe, or, in the case of electromagnetic stirring, during a single solidification event to refine the fusion-zone grains. In WAAM, they are applied layer by layer, so each treated layer becomes the substrate for the next one and is reheated by it. The imposed plastic strain must therefore survive, or be released by, this reheating to produce the intended effect: in Ti-6Al-4V, the strain stored by rolling or hammer peening leads to new β grains when the next layer reheats the material [67,95], and the refinement accumulates through the build rather than being confined to a surface layer [20]. The treatment must also reach the top of a growing wall and be synchronized with the inter-pass temperature, which in turn changes the thermal history of the subsequent layers.

Inter-layer rolling is the most extensively studied deformation-assisted route. Rolling each layer of WAAM steel walls reduced distortion, which was eliminated with a slotted roller, lowered the residual stress, particularly near the baseplate, and refined the grains when the rolled material was reheated by the next pass [26]; in Ti-6Al-4V, rolling each layer refined the centimetre-scale columnar prior-β grains to below 100 µm and weakened both the β and α textures to near random, which Donoghue et al. [67] attributed to new β orientations formed at deformation heterogeneities acting as nuclei on reheating; high-pressure interpass rolling produced equiaxed prior-β grains of 56–139 µm [85], and larger roller radii gave more uniform recrystallization while higher loads increased refinement, with most of the strain generated within 3 mm of the rolled surface [69]; and in 2319 (Al–Cu) walls, vertical inter-pass rolling virtually eliminated distortion and side rolling produced fairly uniform longitudinal compressive stresses [25], while inter-layer rolling at 45 kN raised the ultimate tensile and yield strengths to 314 and 244 MPa, respectively [96]. The principal limitations of rolling are the high contact loads required, the need for a roller path that follows the deposit, and the resulting restriction to simple, accessible geometries such as straight or gently curved walls.

Peening introduces compressive residual stress and plastic strain from the surface. Laser shock peening of WAAM 2319 aluminum converted the tensile residual stress to compressive (maximum about 100 MPa), reduced the average grain size from 59.7 to 46.7 µm, and increased the yield strength by 72% [97]. Because the plastically affected depth is limited, peening is most effective when applied between layers or to critical surfaces, and it has little influence on the bulk columnar structure of thick sections.

Inter-layer friction stir processing imposes severe plastic deformation below the solidus, producing a dynamically recrystallized, fine equiaxed stir zone, fragmenting interdendritic eutectic networks, and closing porosity within the stirred volume [74,98]. Compared with rolling, FSP produces a much finer stir-zone grain size but is limited by tool wear and process forces in high-melting-point alloys, by the requirement that the stir-zone depth exceed the layer thickness to avoid unprocessed interlayer bands, and by exit holes and geometric access; it is therefore currently most mature for aluminum alloys. Pore closure by FSP also depends on the thermal history of each layer: in three-layer Al 4043 walls, the upper layer retained higher porosity (2.90 ± 1.18%) than the bottom layer despite a lower peak FSP surface temperature, which was associated with its lower post-peak cooling rate (−12.3 versus −16.2 °C/s) [99].

Inter-layer or final machining in hybrid additive–subtractive systems removes surface waviness and stair-stepping and provides a flat reference surface for the next layer [10,11,41], but it leaves the as-deposited microstructure and residual-stress state essentially unchanged and adds cycle time.

**Figure 21:**
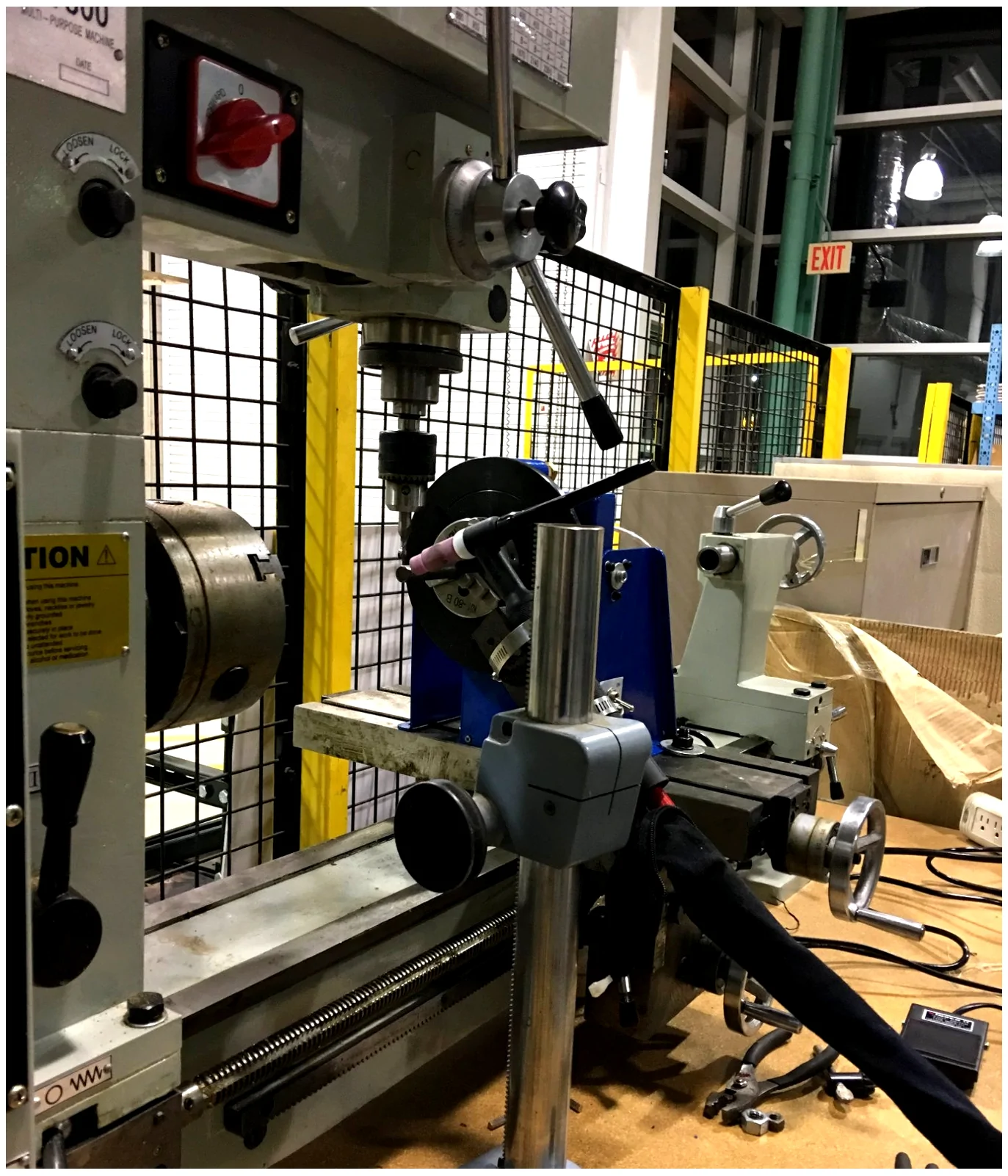
Experimental setup of the hybrid WAAM-FSP Additive–Deformation–Machining (ADM) platform. Original photograph, courtesy of the authors [100]; the same platform was used in [74,101].

The authors’ Additive–Deformation–Machining (ADM) process, also reported as the unified additive–deformation manufacturing process (UAMFSP) [100], is one implementation of the FSP-based route in which GMAW deposition, inter-layer FSP, and CNC milling are performed sequentially within one CNC workcell (Figure 21). The GMAW (MIG) torch is mounted on a multi-purpose CNC machine adjacent to the FSP spindle; after each layer cools to the inter-pass temperature, the FSP tool traverses the bead, and the milling tool can then restore a flat reference surface for the next layer. In ER4043 aluminum walls, infrared thermography showed peak layer temperatures of approximately 870–1000 °C for MIG-only deposition, whereas the MIG + FSP walls maintained more uniform sub-solidus temperature profiles below approximately 400 °C; the mean grain area decreased from approximately 314 µm^2^ to 10.9 µm^2^, and the Vickers microhardness (HV0.2) increased from 52.0 ± 1.3 to 75.8 ± 7.7 [74,101]. These results were obtained on single aluminum walls; residual stress, fatigue performance, and transfer to high-melting-point alloys and complex geometries are not addressed in [74] and remain to be established for this route, which shares the tool-wear, stir-depth, and access limitations of the other FSP-based hybrids in Table 8.

**Table 8:** Comparison of deformation- and machining-based hybrid WAAM strategies. Reported effects are qualitative summaries of the cited studies; magnitudes depend on alloy, geometry, and process conditions.

Strategy	Principal Mechanism	Reported Effects (Alloy)	Main Limitations	Refs.
Inter-layer rolling	Plastic strain in each layer; recrystallization on reheating	Distortion eliminated, lower residual stress, and grain refinement (steel); prior-β grains refined to <100 µm with near-random texture (Ti-6Al-4V); distortion virtually eliminated and higher strength (2319 Al–Cu)	High contact loads; laterally restrained or intersecting features require special roller profiles	[25,26,67,69,85,96]
Peening (e.g., laser shock)	Near-surface compressive stress and plastic strain	Tensile residual stress converted to compressive (≈100 MPa); grain size 59.7 → 46.7 µm; yield strength +72% (Al 2319)	Limited affected depth; little effect on bulk columnar structure	[97]
Inter-layer FSP (including ADM)	Severe plastic deformation below solidus; dynamic recrystallization	Fine equiaxed stir zone; eutectic fragmentation; reduced porosity; lower peak layer temperature and higher hardness (Al alloys)	Tool wear and forces in high-melting-point alloys; stir depth must exceed layer thickness; exit holes; access	[74,98,100]
Inter-layer or final machining	Material removal	Improved dimensional accuracy; flat reference surface	No microstructural or residual-stress benefit; added cycle time	[10,11,41]

From the metallurgical framework developed throughout this review, the ADM process operates through sequential thermal and deformation mechanisms. Each GMAW deposition pass establishes a melt pool governed by heat-source behavior, with solidification conditions determined by the local *G*/*R* ratio and cooling-rate product. The resulting columnar microstructure is then disrupted by the FSP pass, which imposes high plastic strain in the stir zone and activates dynamic recrystallization. The Hall–Petch relationship in Table 5 provides a qualitative basis for the associated strengthening, although the hardness increase after FSP may also reflect fragmentation and redistribution of the eutectic network and should not be attributed to grain refinement alone [74].

Because the stir zone is returned to a recrystallized state after each layer, FSP-based hybrids may reduce build-to-build microstructural variability relative to plain WAAM; this has not yet been quantified statistically and should be demonstrated before it is used in qualification arguments (Section 9.2).

Cold Metal Transfer is an important process development in GMAW-WAAM, but its advantages are conditional rather than universal. By reducing H through mechanically assisted and current-minimized droplet detachment, CMT can shift the operating condition toward lower heat accumulation, reduced spatter, and improved bead regularity, which suits thin-walled aluminum and steel structures. Its limitation is that wire feed and arc power remain coupled, so heat input and deposition rate cannot be set fully independently (Section 4.2). PAW and GTAW provide that independence and greater control of arc characteristics, and PAW-based deposition of Ti-6Al-4V has reached about 2 kg/h [46], whereas conventional GMAW remains preferable when deposition rate outweighs geometric accuracy. The process should therefore be selected against explicit criteria: the alloy and its sensitivity to oxidation, porosity, and cracking; the required deposition rate; the wall thickness and geometric tolerance; the permissible heat input; and the required surface quality. Through Equations (10) and (11), lower-heat-input variants such as CMT Advanced [51] are expected to favor finer structures and, via the Hall–Petch relationship in Table 5, higher strength; this link must nevertheless be verified for each alloy, because transformation products and precipitation state also contribute to strength.

The limiting factor for CMT adoption is equipment cost, although this barrier is expected to decrease progressively as the technology matures and as the competitive landscape in welding equipment manufacturing responds to growing demand from the AM sector. The technical case for CMT as a preferred GMAW mode is strongest in WAAM applications where low heat input, reduced spatter, dimensional accuracy, and microstructural control are primary concerns.

### 10.3. Future Research Priorities

Despite the considerable body of literature reviewed herein, several important knowledge gaps remain:

- **Closed-loop adaptive process control**: An industrially deployable architecture for real-time correction of geometric deviations and microstructural anomalies during WAAM is not yet available at broad commercial readiness. Machine-learning approaches trained on large WAAM datasets hold promise for multi-variable parameter optimization, but training-data requirements, sensor reliability, and generalizability across alloy systems remain open challenges.
- **Validated 3D thermal models for complete builds**: While single-bead and single-layer thermal models are well developed, validated three-dimensional thermo-mechanical models of complete WAAM builds, including complex path geometries and 50–200 layer structures, are not yet routinely available as engineering tools.
- **Fatigue performance characterization and design allowables**: The fatigue data necessary to populate structural design allowables for WAAM components are insufficient for most alloy systems. The sensitivity of WAAM fatigue performance to microstructural heterogeneity, surface waviness, porosity, and residual stress demands systematic, statistically robust S-N curve development across multiple alloy systems before broad aerospace certification is achievable.
- **Multi-material and functionally graded WAAM**: The ability to spatially vary chemistry within a single WAAM build has been demonstrated experimentally, but it lacks the systematic parametric understanding required for reliable engineering application. The interfacial microstructure and bond quality at composition transitions require particular attention.
- **Standardization and qualification**: The development of process qualification and component certification standards for WAAM, analogous to those established for more mature manufacturing routes, remains one of the most critical non-technical challenges for industrial adoption. Standardization bodies are engaged, but progress remains slower than the underlying technology development.

## 11. Conclusions

This review demonstrates that WAAM is best understood not simply as a high-deposition-rate AM process, but as a layer-wise transformation of traditional arc welding science in which conventional heat-source, solidification, and defect-formation principles must be reinterpreted under repeated thermal cycling.

This review has examined the adaptation of traditional arc welding processes for Wire Arc Additive Manufacturing (WAAM), integrating governing physical principles, process-microstructure relationships, and defect-formation mechanisms into a quantitative framework. This review yields the following principal conclusions:

- The *G*/*R* ratio framework from classical solidification theory, linking the temperature gradient G and solidification rate R at the melt-pool boundary, provides a unifying quantitative tool for interpreting WAAM microstructural outcomes. The transition from columnar-dendritic to equiaxed-dendritic solidification occurs approximately when *G*/*R*^1/2^ < *C_CET_*, but promoting this transition in WAAM usually requires active intervention because directional heat extraction often favors columnar growth.
- WAAM and traditional fusion welding share closely related governing physics, including melt-pool thermodynamics, Marangoni convection, solidification theory, and solid-state transformation kinetics. However, WAAM differs critically because accumulated thermal cycling, repeated reheating, and progressive heat accumulation generate base-to-top microstructural gradients, banded precipitation or dissolution behavior, residual stress accumulation, and evolving *G*/*R* conditions that have no direct equivalent in conventional single-pass welding.
- Among the principal arc-based WAAM processes, GMAW remains the industrial workhorse for large-volume structural deposition; GTAW provides high arc stability and shielding control for precision and reactive-metal applications; and PAW offers high energy density and reduced distortion potential at the cost of greater equipment complexity.
- Among GMAW variants, Cold Metal Transfer (CMT) provides controlled droplet detachment, low heat input, reduced spatter, and improved bead regularity; because wire feed and arc power remain coupled in CMT, GTAW and PAW are preferable when heat input and deposition rate must be controlled independently, as for titanium alloys, so the choice of process depends on alloy, deposition rate, geometry, and surface-quality requirements. The mechanically assisted droplet detachment of CMT is related to the balance between surface tension, electromagnetic pinch force, transition current, and per-cycle energy, as described by Equations (7)–(10). The mechanically assisted droplet detachment of CMT, combined with current minimization during short-circuiting, explains its ability to reduce heat input and distortion relative to conventional GMAW transfer modes in many WAAM applications.
- Porosity in aluminum WAAM is governed by hydrogen dissolution and rejection during solidification, described by Sieverts’ law (Equation (24)), and by pore escape or entrapment, described by the Stokes velocity criterion (Equation (25)). These relationships explain why an intermediate heat-input window is often required: insufficient heat input may leave hydrogen-bearing contamination, whereas excessive heat input enlarges the melt pool, promotes turbulence, and increases pore-entrapment probability.
- The principal governing relationships linking process parameters to microstructure and properties in WAAM include the arc power and linear heat input definitions (Equation (1)), the Goldak double-ellipsoidal heat-source model (Equations (15) and (16)), the constitutional undercooling criterion (Equation (18)), the dendrite arm spacing relationship (Equation (20)), the Hall–Petch strength–grain size relationship summarized in Table 4, and the thermal strain and residual stress equations (Equations (21) and (22)). Together, these relationships form a process–microstructure–property framework for WAAM design and defect interpretation.
- Open research priorities for WAAM include closed-loop adaptive process control enabled by in-situ monitoring and machine learning; validated three-dimensional thermo-mechanical models for complete builds; systematic fatigue characterization and design allowables across alloy systems; reliable strategies for multi-material and functionally graded WAAM; and accelerated development of international qualification standards [102,103].

The future industrial maturity of WAAM will depend not only on increasing deposition rate or component size, but on controlling the coupled thermal, metallurgical, mechanical, and qualification challenges that arise when traditional welding processes are transformed into layer-wise additive manufacturing systems.

## Figures and Tables

**Table 1:** Process comparison between WAAM and laser-based AM processes [30,31,32].

Parameter	WAAM	Laser-Based AM
Power Source	Standard welding power supply	High-power laser with complex optics
Energy Efficiency	>80% (source + transfer)	~20% laser; up to 40% coupling
Deposition Rate	1–10 kg/h; typically 2–4 kg/h	Tens of grams/h for powder bed
Material Utilization	~100%; wire is fully consumed	Partial; powder recycling required
Component Size	Large up to several meters	Limited by chamber/optical envelope
Geometric Complexity	Low to medium	High (powder bed)
Surface Finish (Ra)	10–50 µm; post-machining usually needed	<10 µm; often usable as-built
Equipment Cost	Low–moderate	High
Operating Cost	Low	High (powder, gas, maintenance)
Residual Stress	High due to cyclic arc heat input	Moderate–high (rapid cooling)
References	[7,30,31,32]	[5,33,34]

**Table 2:** Author-compiled comparative assessment of GMAW, GTAW, and PAW for WAAM applications, synthesized from the cited literature [32].

Criterion	GMAW	GTAW	PAW
Arc Energy Density	Medium	Medium–low	High (highest of three)
Deposition Rate	2–4 kg/h	0.5–2 kg/h	1–4 kg/h
Deposit Quality	Good	Excellent	Excellent
Distortion Risk	Moderate	Low	Minimal
Equipment Cost	Low	Low–medium	High
Reactive Metals	Good with shielding	Excellent	Excellent
Automation Ease	High	Moderate	High
Best Application	Large structural, high throughput	Precision, reactive metals	High-speed, quality-critical
Wire feed and heat input	Coupled (wire is the electrode; synergic control)	Independent (separate filler wire)	Independent (separate filler wire)

**Table 3:** Author-compiled summary of GMAW metal-transfer modes and their suitability for WAAM applications [47,48,49].

Mode	Mechanism	Current Range	Heat Input	AM Suitability
Short Circuit (SCT)	Wire contacts melt pool 90–200×/s; arc extinguishes at each contact	Low	Low	Good low heat; moderate spatter
Globular	Large droplets at wire tip fall under gravity and electromagnetism	Low–mid	Medium	Poor spatter, irregular beads
Spray Arc	Fine droplets sprayed at high frequency from electrode	High	High	Poor excessive heat, grain coarsening
CMT	Wire feed and current synchronized; wire retracts to deposit at up to 70 Hz	Very low	Very low	Excellent minimal spatter, superior quality; higher cost

**Table 4:** Summary of governing equations for WAAM process–microstructure–defect relationships.

Equation	Parameters	Significance in WAAM
P=ηVI; H=ηVI/v	*η*: arc thermal efficiency; *V*: voltage (V); *I*: current (A); *v*: travel speed (mm/s)	*P*: effective arc power (W), input to heat-source models; *H*: linear heat input (J/mm), governs melt-pool size, cooling rate, and remelting depth
${\bar{d}}^{\;n}-{\bar{d}}_{0}^{\;n}=K_{0}\;t\;exp\left(-\frac{Q_{g}}{R_{g}T}\right)$	$\bar{d}$, $\bar{d}$_0_: current and initial mean grain size (m); n: growth exponent (2 for ideal growth); *K*_0_: kinetic constant (m^n^/s); *Q_g_*: activation energy for grain growth (J/mol); *R_g_*: gas constant (J·mol^−1^·K^−1^); *T*: temperature (K); *t*: time at *T* (s)	Isothermal grain-growth kinetics; applied to repeated reheating by integrating over the thermal history
$\sigma_{y}=\sigma_{0}+k_{y}d^{-1/2}$	*σ*_0_: friction stress; k_y_: Hall–Petch slope; *d*: grain diameter (m)	Yield strength improvement from grain refinement
$J_{H}=-D_{H}\frac{\partial C_{H}}{\partial x}\approx D_{H}\frac{\Delta C_{H}}{\delta }$	*D_H_*: hydrogen diffusivity (m^2^/s); *C_H_*: hydrogen concentration; *δ*: diffusion boundary-layer thickness (m)	Hydrogen flux driving porosity formation in melt pool
$\varepsilon_{th}=\alpha_{CTE}\;\Delta T$	*α_CTE_*: coefficient of thermal expansion (K^−1^); *ΔT*: temperature change (K)	Thermal strain driving residual stress and distortion
$G/R\geq \Delta T_{0}/D_{L}$	*G*: thermal gradient (K/m); *R*: solidification rate (m/s); *ΔT*_0_: equilibrium solidification range (K); *D_L_*: solute diffusivity in liquid (m^2^/s)	Planar-front stability criterion; decreasing *G*/*R* gives planar → cellular → columnar-dendritic → equiaxed growth
$\lambda_{1}=a{\left(G\cdot R\right)}^{-n}$	*λ*_1_: primary dendrite arm spacing (m); *G*·*R*: cooling rate (K/s); a, n: alloy constants	Predicts primary dendrite arm spacing; governs inter-dendritic segregation

**Table 5:** Principal defects in WAAM: mechanisms, governing parameters, and mitigation strategies [16,32,44,59,60,61,65,66,70].

Defect	Mechanism	Governing Parameters	Mitigation Strategies
Porosity	H rejection during solidification; turbulent melt pool entraining gas	Welding current, travel speed, shielding gas, wire cleanliness	Wire/substrate cleaning; CMT or SCT; optimize *G*/*R* ratio
Solidification Cracking	Tensile stress on semi-solid film; high solute segregation at grain boundaries	Alloy composition, cooling rate, restraint	Grain refiners; reduce heat input; modify alloy chemistry
Residual Stress	Differential thermal contraction under structural constraint	Heat input, path strategy, inter-pass temperature	Inter-pass cooling; stress relief; contour path; peening
Delamination	Insufficient inter-layer remelting; poor metallurgical bonding	Inter-pass temperature, overlap distance, heat input	Optimize inter-pass temperature window; overlap control
Columnar Grain Coarsening	Epitaxial growth along build direction driven by thermal gradient G	*G*/*R* ratio, cooling rate, alloy SFE	Grain refiners; arc oscillation; inter-pass rolling; CMT-P
Geometric Deviation	Bead instability at arc start/end; heat accumulation changing bead profile	Travel speed ramp-up/down, inter-pass temperature	Adaptive start/end correction; dwell periods; hybrid machining

**Table 6:** Traceable quantitative results from primary WAAM studies. Values are transcribed from the text or tables of the cited study for the stated condition; no values were digitized from figures.

Alloy (Feedstock)	Process	Controlled Variable	Reported Result (Condition)	Ref.
Aluminum alloy	Hot-wire arc additive manufacturing	Hot-wire current	Pores clustered at interlayers; porosity minimum at 100 A; relative density 96.8% (0 A) and 99.6% (100 A); porosity increased again above 100 A	[44]
ER4043 aluminum	GMAW (MIG) and MIG + inter-layer FSP (ADM/UAMFSP)	Inter-layer FSP (without/with)	Peak layer temperature ≈ 870–1000 °C (MIG) vs. sub-solidus profiles below ≈ 400 °C (MIG + FSP); mean grain area ≈ 314 µm^2^ vs. 10.9 µm^2^; HV0.2 52.0 ± 1.3 vs. 75.8 ± 7.7	[74]
Low-alloy high-strength steel (metal-cored wire)	Arc-based WAAM	Inter-pass temperature (150, 350, 600 °C)	No obvious microstructural change between 150 and 350 °C; no significant texture; highest UTS at 350 °C; wall collapse at 600 °C; 350 °C identified as upper limit	[63]
AISI 420 martensitic stainless steel	Conventional GMAW, CMT, CMT Advanced, CMT Pulse	Transfer-mode variant	CMT Advanced: lowest heat input and best wall-height performance; CMT Pulse: opposite extreme; CMT and conventional GMAW similar	[51]
IN718 on S275 steel substrate	WAAM hybrid component	Post-deposition heat treatment (AB, ST 1080 °C/1 h, STA)	Strong ⟨002⟩ texture parallel to BD in all states; Laves retained near interface after ST; Nb microsegregation eliminated and hardness decreased after ST	[75]
ER309LSi stainless steel (thick walls, 8 layers)	CMT and CMT Pulse	Transfer variant; interlayer idle time 0–300 s	At equal wire feed rate CMT Pulse input a higher power; with 0 s idle time the area above 900 °C in the last layer was 1013 mm^2^ (CMT Pulse) vs. 400.8 mm^2^ (CMT); mean interlayer temperature 15.0% higher for CMT Pulse; idle time controlled heat accumulation for both	[52]
Inconel 625	GTAW-based AM (wall)	Location in the wall	Cellular grains near substrate, upward columnar dendrites in layer bands, transition to equiaxed grains at top; Nb and Mo segregation increased with height; PDAS correlated with Laves content; highest microhardness and tensile strength near substrate	[76]
ER1100, ER4043, ER4943, ER4047, ER5356 aluminum	GMAW-based 3-D printing	Filler alloy	Porosity < 2% for all alloys, highest for ER5356; 4000-series alloys best for bead width, porosity, strength, and defect sensitivity	[77]
316L stainless steel	GMAW-based AM	Reheating by subsequent layers	δ and σ phases transformed to fine vermicular morphologies within austenite dendrites after the fourth layer; tensile properties comparable to wrought 316L	[78]

AB, as-built; ST, solution-treated; STA, solution-treated and aged; BD, build direction; UTS, ultimate tensile strength; FSP, friction stir processing.

**Table 7:** Anchor descriptors used for the qualitative ratings in Figure 19.

Criterion	Definition	Low (1–3)	Intermediate (4–6)	High (7–10)
Porosity control	Ease of achieving low porosity under typical WAAM conditions	Porosity is a principal limitation (e.g., high hydrogen pickup or volatile elements)	Controlled through feedstock and shielding cleanliness and a heat-input window	Porosity rarely limiting
Surface quality	As-deposited bead regularity and waviness	Irregular beads, humping, or spatter common	Regular beads with standard parameter optimization	Consistently regular beads
Deposition rate	Achievable productivity with the process variants suited to the alloy	Restricted to low-heat-input or GTAW/PAW variants	Moderate rates with GMAW or CMT variants	High rates with GMAW
Cost efficiency	Feedstock, shielding, and processing cost relative to component value	High feedstock cost and/or inert chamber required	Moderate	Low-cost wire and local shielding
Fatigue performance	Expected fatigue behavior of machined as-deposited material	Strongly limited by defects or microstructure	Adequate with post-processing	Favorable in the as-deposited condition
Residual-stress management	Ease of limiting distortion and residual stress	Distortion or cracking frequently limiting	Manageable with inter-pass control and fixturing	Rarely limiting
Grain refinement	Ease of avoiding coarse columnar structures	Strong epitaxial columnar growth persists	Partial refinement by thermal cycling or process control	Refined or equiaxed structures readily achieved

## Data Availability

No new data were created or analyzed in this study. Data sharing is not applicable to this article.

## Abbreviations

**Abbreviation**	**Definition**
AM	Additive Manufacturing
WAAM	Wire Arc Additive Manufacturing
DED	Directed Energy Deposition
CMT	Cold Metal Transfer
MIG	Metal Inert Gas Welding
MAG	Metal Active Gas Welding
LPBF	Laser Powder Bed Fusion
DRX	Dynamic Recrystallization
*G*	Temperature Gradient (K/m)
UTS	Ultimate Tensile Strength
SFE	Stacking-Fault Energy (mJ/m^2^)
HIP	Hot Isostatic Pressing
FSP	Friction Stir Processing
SCT	Short Circuit Transfer
HAZ	Heat-Affected Zone
GMAW	Gas Metal Arc Welding
GTAW	Gas Tungsten Arc Welding
PAW	Plasma Arc Welding
TIG	Tungsten Inert Gas Welding
P; H	Effective Arc Power (W); Linear Heat Input (J/mm)
RSM	Response Surface Methodology
ANOVA	Analysis of Variance
*R*	Solidification Rate (m/s)
HV	Vickers Hardness
EBSD	Electron Backscatter Diffraction
CNC	Computer Numerical Control
LSP	Laser Shock Peening
